# A General One-Step Synthesis of Alkanethiyl-Stabilized Gold Nanoparticles with Control over Core Size and Monolayer Functionality

**DOI:** 10.1021/acs.chemmater.3c01506

**Published:** 2023-07-17

**Authors:** Stefan Borsley, William Edwards, Ioulia K. Mati, Guillaume Poss, Marta Diez-Castellnou, Nicolas Marro, Euan R. Kay

**Affiliations:** EaStCHEM School of Chemistry, University of St Andrews, North Haugh, St Andrews KY16 9ST, U.K.

## Abstract

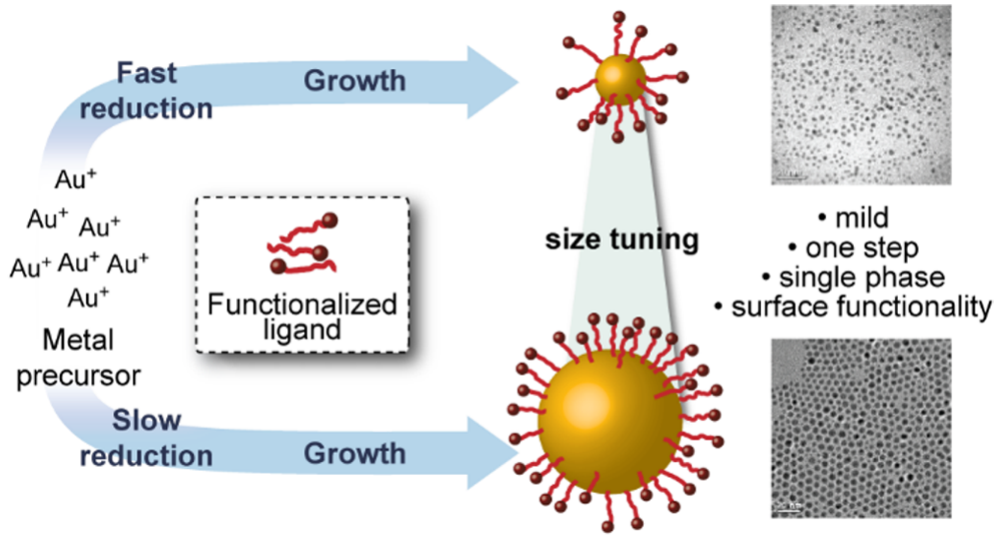

In spite of widespread interest in the unique size-dependent properties and consequent applications of gold nanoparticles (AuNPs), synthetic protocols that reliably allow for independent tuning of surface chemistry and core size, the two critical determinants of AuNP properties, remain limited. Often, core size is inherently affected by the ligand structure in an unpredictable fashion. Functionalized ligands are commonly introduced using postsynthesis exchange procedures, which can be inefficient and operationally delicate. Here, we report a one-step protocol for preparing monolayer-stabilized AuNPs that is compatible with a wide range of ligand functional groups and also allows for the systematic control of core size. In a single-phase reaction using the mild reducing agent *tert*-butylamine borane, AuNPs that are compatible with solvents spanning a wide range of polarities from toluene to water can be produced without damaging reactive chemical functionalities within the small-molecule surface-stabilizing ligands. We demonstrate that the rate of reduction, which is easily controlled by adjusting the period over which the reducing agent is added, is a simple parameter that can be used irrespective of the ligand structure to adjust the core size of AuNPs without broadening the size distribution. Core sizes in the range of 2–10 nm can thus be generated. The upper size limit appears to be determined by the nature of each specific ligand/solvent pairing. This protocol produces high quality, functionally sophisticated nanoparticles in a single step. By combining the ability to vary size-related nanoparticle properties with the option to incorporate reactive functional groups at the nanoparticle–solvent interface, it is possible to generate chemically reactive colloidal building blocks from which more complex nanoparticle-based devices and materials may subsequently be constructed.

## Introduction

Metal nanoparticles (NPs) have generated much excitement as a result of their distinct properties, which are dependent on the core material, size, and shape, making them of interest for an ever-expanding range of applications.^1−3^ Commonly, the inorganic core is stabilized by a surface-bound monolayer of molecular ligands, as exemplified by the archetypal alkanethiyl-stabilized gold nanoparticle (AuNP).^4,5^ Defining the interface between the nanoparticle core and surrounding matrix, the ligand shell is also a critical determinant of numerous properties, including solvent compatibility, catalytic activity, nanoparticle–molecule interactions, and optical and electronic behavior.^6−10^ Furthermore, surface-bound ligands that incorporate reactive sites provide a handle for conjugating additive functionality or appending nanoparticles to other components in postsynthesis manipulations.^11−14^ Consequently, size-controlled synthesis of nanoparticle populations with narrow size distributions is critical for tuning nanoparticle properties and is, therefore, a long-standing central challenge in nanochemistry. However, the majority of methods focus only on controlling features of the metallic core, typically stabilized by nonfunctionalized ligands that are inadequate for many applications.^15^ Synthesis strategies that easily allow for independent tuning of both the core and the ligand parameters will accelerate the systematic and efficient development of nanoparticle-based applications.

**Figure 1:**
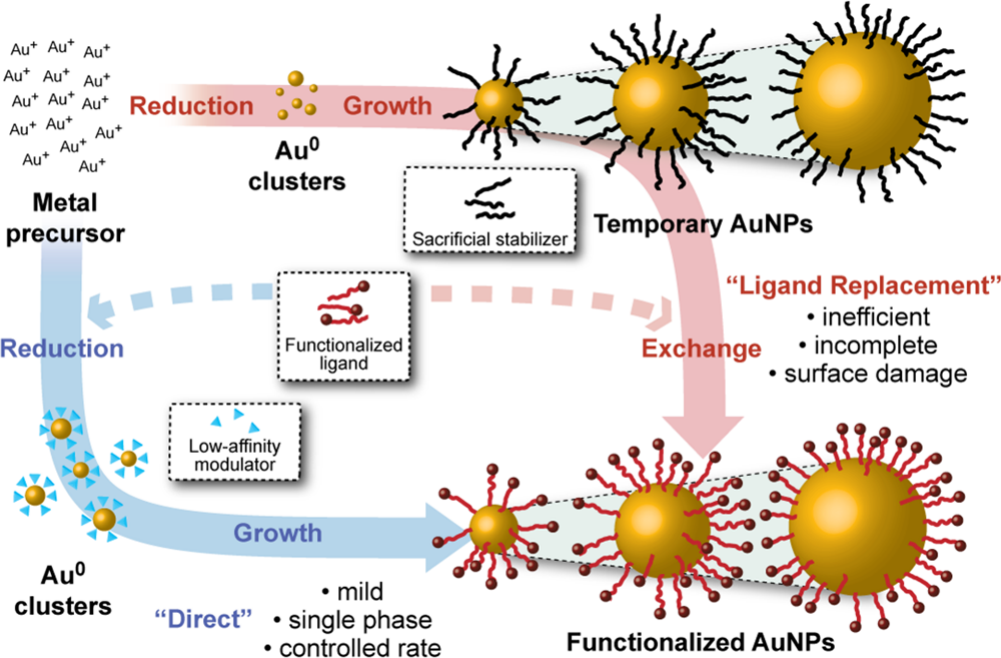
Schematic representation of synthetic strategies for producing functionalized monolayer-stabilized AuNPs. The “ligand replacement” route indicated by the red arrow involves the size-controlled synthesis of functionalized nanoparticles via intermediate particles coated in a sacrificial species, followed by a place-exchange with the desired ligand. The blue arrow indicates the potentially more efficient “direct” size-controlled synthesis of functionalized AuNPs, in which the desired ligand is present from the outset. For either route, each process (cluster formation, nanoparticle growth, ligand exchange) can entail a number of different atomic-level mechanisms, depending on the reaction conditions as well as the degree of overlap/separation of nucleation and growth processes.

Since the first practical synthesis of alkanethiyl-stabilized AuNPs by Brust and Schiffrin,^16^ there have been several important advances in the synthetic methodology.^15,17,18^ However, size control remains a significant challenge, particularly when bespoke ligand shells are required. Monolayer functionalization is most commonly achieved by postsynthesis ligand replacement (Figure 1, red route), where the desired ligand is exchanged onto the nanoparticle surface at the expense of a sacrificial stabilizer. When replacing one alkanethiol with another,^19−23^ often the only significant driving force is mass action; thus, exhaustive exchange can be hard to achieve.^24,25^ Ripening or etching processes can also be triggered, which are particularly deleterious to the predictable control of nanoparticle size. Replacing weakly bound sacrificial stabilizers,^15^ such as phosphorus- or nitrogen-based ligands,^26−34^ citrate anions,^35−41^ and alkyammonium surfactants,^39,40,42,43^ can circumvent some of these limitations. However, challenges include the ability to maintain the colloidal stability of the temporary nanoparticle product and purification from the often-required large excesses of the weak stabilizers. Efficient ligand exchange also requires matching the solvent compatibility of the incoming and outgoing stabilizers, thus limiting the range of physicochemical characteristics accessible from each temporary nanoparticle intermediate. From a practical perspective, all ligand-exchange procedures introduce additional steps of nanoparticle manipulation and purification, which has a consequent impact on time and materials efficiency and increases the opportunity for unanticipated issues such as irreversible particle agglomeration.

Therefore, robust protocols that allow a variety of functionalized surface-stabilizing ligands to be directly incorporated during the nanoparticle growth process while also allowing control over the size and dispersity of the core population (Figure 1, blue route) are attractive. However, the direct synthesis of functionalized metal nanoparticles remains underdeveloped. Even minor structural changes to the ligand often result in unanticipated changes to the nanoparticle size distribution,^44^ and few protocols have proven to be robust to more significant changes, such as to the reaction solvent, which would provide compatibility with a broad ligand structural scope.^10,15,17,18^ Similarly, generalizable principles for predictable control over nanoparticle size independent of the ligand structure have not yet been identified; typically, interconnected adjustments to several parameters are required.^45^ Furthermore, the chemical stability of ligand functional groups under the reducing conditions that are required for metal nanoparticle synthesis must always be considered.

To address the unmet need for generalizable synthetic approaches for the direct synthesis of functionalized gold nanoparticles independent of ligand structural details, we investigated the ligand scope of the single-phase AuNP synthesis protocol introduced by Stucky and co-workers.^46,47^ We show that this method can be adapted to create, in a single synthetic step, AuNPs a with narrow size distribution, stabilized with a variety of functionalized ligands and with solvent compatibilities ranging from apolar organic solvents to water. Detailed characterization of the AuNP-bound ligand shell demonstrates single-component monolayers with functionalities unaffected by the synthetic procedure. Significantly, we demonstrate that adjusting just one parameter, the rate of addition of the reducing agent, serves as a general strategy for controlling the AuNP core size, irrespective of the ligand molecular structure. This methodology can be used to create size-controlled AuNPs that are stabilized by chemically reactive ligands, thereby opening the door to efficient divergent routes for a wide range of surface functionalities via standardized nanoparticle preparation and postsynthesis ligand modification protocols,^11−14^ all which avoid ligand-exchange procedures.

The AuNP synthesis described by Stucky^46^ is an attractive starting point for the direct synthesis of a variety of functionalized nanoparticles in non-aqueous solvents. The reducing agent (*tert*-butylamine borane complex, TBAB) is mild compared to other commonly employed reductants such as NaBH_4_, which is traditionally employed in the popular Brust–Schiffrin method.^16^ These milder conditions should allow for compatibility with ligands that may be susceptible to reduction. By employing an organic-soluble gold precursor (AuClPPh_3_), the single-phase Stucky protocol does not require any phase-transfer additives for producing AuNPs coated with hydrophobic ligands; only the gold complex, the reducing agent, and the ligand are included in the reaction mixture, bringing significant advantages for the isolation of pure nanoparticles stabilized by homogeneous single-component monolayers. Importantly, in optimized procedures using simple alkanethiol ligands such as dodecanethiol, this method is capable of achieving excellent size dispersity.^46^ Compatibility with a range of solvents, including EtOH, CHCl_3_, and benzene,^46^ and the preparation of nanoparticles that are stabilized by amphiphilic mixed-ligand monolayers^48^ suggest that the synthesis of functionalized nanoparticles with widely differing solvent compatibilities should be possible. However, this methodology has only been applied to a limited number of unfunctionalized alkanethiols^46,47,49^ and only very rarely to directly incorporate ligands of modest structural complexity.^44,48^ Solvent polarity, temperature, and reagent concentration have all been observed to influence the particle size distribution,^46,47,49^ but generalizable relationships have not emerged. For example, increasing the stoichiometric ratio of stabilizing ligand to gold has been reported to lead to either smaller^50^ or larger^49^ sizes under otherwise very similar conditions, and the effect of temperature is equally unpredictable.^47,49^ We set out to extend the structural scope of AuNP-stabilizing monolayers that may be directly produced using this approach and to develop general principles for varying nanoparticle core size irrespective of the ligand structure.

## Materials and Methods

**Chart 1:**
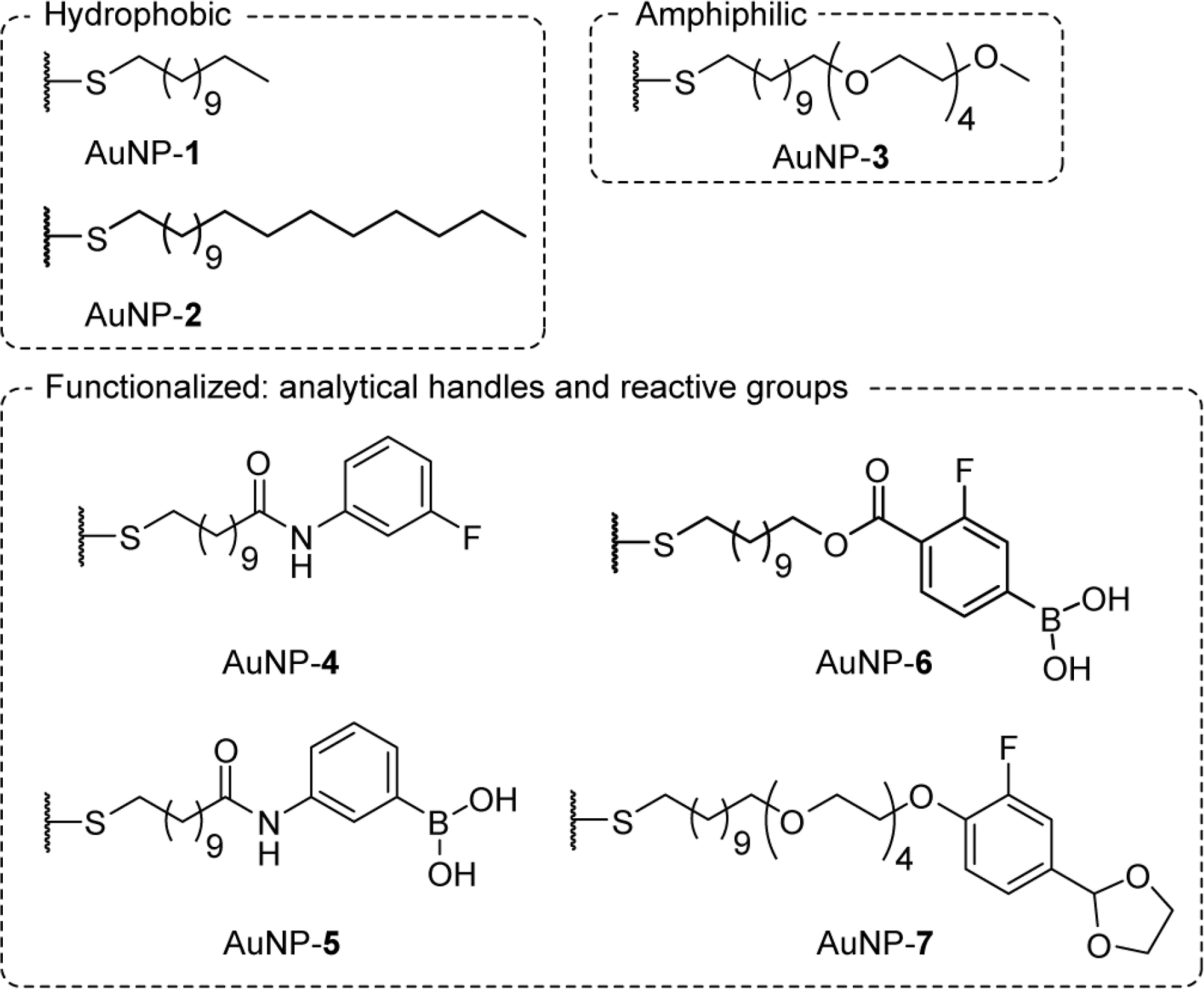
Ligand Structural Scope for Single-Component Monolayer-Stabilized AuNPs

The structures of all of the ligands investigated are shown in Chart 1. Experimental details for the preparation and characterization of the ligand precursors can be found in the Supporting Information.

Nanoparticle syntheses were typically carried out on a reaction scale of 20–25 mg (40–50 μmol) of gold precursor, for which a 10 mL two-neck round-bottom flask was used.

### Standard Procedure for Nanoparticle Synthesis: Instant Addition of Reducing Agent

AuPPh_3_Cl (1 mol equiv) and the ligand precursor (thiol or disulfide, 1.2 mol equiv in terms of sulfur) were dissolved in the reaction solvent to give [Au] ≈ 16.7 mM. The mixture was heated to 55 °C while being stirred vigorously. *tert*-Butylamine borane complex (10 mol equiv) in the reaction solvent (0.5 M) was then added rapidly by syringe to give a final concentration of [Au] ≈ 16 mM. Stirring was continued at 55 °C for 2 h and then at room temperature for a further 16 h.

### Standard Procedure for Nanoparticle Synthesis: Slow Addition of Reducing Agent

AuPPh_3_Cl (1 mol equiv) and the ligand precursor (thiol or disulfide, 1.2 mol equiv in terms of sulfur) were dissolved in the reaction solvent to give [Au] ≈ 16.7 mM. The mixture was heated to 55 °C while being stirred vigorously. *tert*-Butylamine borane complex (10 mol equiv) in the reaction solvent (0.5 M) was then added using a syringe pump at a controlled rate to achieve total addition over the intended time period. Heating was continued at 55 °C for a total of 2 h (including the time taken for addition of the reducing agent); then, heating was removed and stirring continued at room temperature for a further 16 h.

### Nanoparticle Isolation and Characterization

Nanoparticles were isolated by ensuring complete precipitation with a nonsolvent, leaving a colorless supernatant, which was carefully decanted. The solid residue was resuspended in a good solvent by sonication to ensure any material adhering to the vessel walls was redispersed prior to analysis by transmission electron microscopy (TEM). Consequently, the reported size distributions reflect only the outcome of the synthesis process, independent of any alteration of size distributions that might be possible via optimized purification methods such as size-selective precipitation or size-exclusion chromatography.

Details of the analytical experiments can be found in the Supporting Information.

## Results and Discussion

### Extending the Ligand Scope of the Direct Nanoparticle Synthesis Protocol

**Figure 2:**
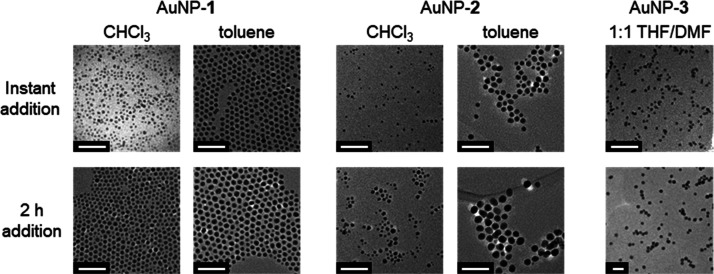
Representative TEM images for AuNPs stabilized by hydrophobic (AuNP-**1** and AuNP-**2**) and amphiphilic (AuNP-**3**) surface-bound ligands according to the reaction solvent and the rate of addition of the reducing agent. Scale bars: 40 nm. Further images and histograms representing each size distribution can be found in the Supporting Information (AuNP-**1**: Figures S1–S7; AuNP-**2**: Figures S8–S11; AuNP-**3**: Figures S12 and S13).

Initially, we repeated the synthesis of dodecanethiyl-coated AuNPs in order to verify that we could reliably reproduce the previously reported results.^46^ In a typical procedure (see Materials and Methods for details), TBAB was added as a solution in a single portion to a mixture of the gold precursor and the ligand at 55 °C. Then, the reaction was maintained at this temperature for 2 h, followed by 16 h at room temperature. Nanoparticles were isolated by one round of precipitation and washing with a nonsolvent. By this method, we obtained AuNP-**1** with ⟨*d*⟩ = 2.71 ± 0.64 nm (24% dispersity) when we carried out the synthesis in CHCl_3_ and ⟨*d*⟩ = 5.70 ± 0.51 nm (9%) when we carried out the synthesis in toluene (Figures 2, S1, and S6). Pleasingly, these results are in reasonably good agreement with those reported by Stucky and co-workers^46^ under similar conditions (3.5 ± 0.3 nm (9%) in CHCl_3_ and 6.2 ± 0.3 nm (5%) in benzene).^51^ It has been demonstrated that the alkanethiol chain length can have a moderate, but solvent-specific, effect on AuNP size: larger particles are observed with longer alkyl chain lengths across the range C_6_–C_16_ for syntheses in benzene, while very little change is observed for particles prepared in CHCl_3_.^47^ Extending this series to the C_18_ analogue AuNP-**2**, we observe a continuation of these trends, with preparation in toluene giving slightly larger sizes (⟨*d*⟩ = 7.26 ± 1.24 nm, 17%; Figures 2 and S10), while using CHCl_3_ as the reaction solvent gives a similar outcome (⟨*d*⟩ = 3.12 ± 0.72 nm, 23%; Figures 2 and S8) compared to the C_12_ baseline.

Having confirmed a baseline protocol, we sought to expand the structural scope of the ligands that could be directly incorporated by using this method (Chart 1). Monolayer-stabilized metal nanoparticles have received significant attention for numerous biological and biomedical applications, including imaging and drug delivery.^1−3^ Such applications nearly always necessitate water solubility and, as such, represent a significant challenge to synthetic methods optimized for apolar solvents. Therefore, we investigated the production of water-soluble AuNPs stabilized by an amphiphilic tetra(ethylene glycol)-alkanethiyl ligand (AuNP-**3**). In this case, it was synthetically more convenient to prepare and purify the ligand precursor as a disulfide dimer rather than as a thiol. Exploiting the reversible redox interconversion between thiols and disulfides is a common strategy for controlling sulfur reactivity during synthetic manipulation and purification.^52^ Disulfide precursors have been used directly in AuNP synthesis procedures using borohydride reductants, where the disulfide bond is rapidly cleaved under the reaction conditions.^24,53−59^ Borane reducing agents should likewise be capable of reducing disulfides,^44^ and we found similar results for several examples reported here, irrespective of whether the ligand precursor was introduced as a disulfide or as a thiol. Although the amphiphilic disulfide **3**_2_ is not itself soluble in water, when bound in the AuNP-stabilizing monolayer, only the hydrophilic tetra(ethylene glycol) segment is exposed to the surrounding matrix, thus imparting excellent water solubility on AuNP-**3**. Producing high quality nanoparticles requires that the reaction solvent effectively solubilizes both the ligand precursor and the nanoparticle products. We found that a mixture of THF and DMF (1:1 *v*/*v*) meets these requirements, giving AuNP-**3** with ⟨*d*⟩ = 3.72 ± 0.58 nm (16%; Figures 2 and S12).

The ubiquity of amine and carboxylic acid functional groups across multiple compound categories, together with their numerous facile coupling protocols, has made amides one of the most common linkages for constructing α,ω-functionalized alkanethiol or disulfide monolayer precursors.^52^ Meanwhile, ^19^F NMR spectroscopy has played a critical role in our efforts to achieve nondestructive in situ characterization of nanoparticle-bound molecular structures and real-time reaction tracking.^60−66^ Pleasingly, the protocol could be adapted to produce AuNP-**4** stabilized by a monolayer of fluorine-substituted amide (⟨*d*⟩ = 3.86 ± 0.39 nm, 10%; Figure S14), in this case by using THF/MeOH (10:1 *v*/*v*) as the reaction solvent.

Only a limited range of chemical functionalities is compatible with the conditions required for nanoparticle preparation. Furthermore, the necessity to prepare a new alkanethiol or disulfide for each desired monolayer structural change is synthetically tedious. Nanoparticle “building-block” strategies are far more versatile, wherein carefully chosen reactive groups are incorporated during nanoparticle synthesis, thereby allowing for divergent postsynthesis modification to produce any number of monolayer structural variations starting from a single nanoparticle starting point.^12,14^ For example, we have developed on-nanoparticle dynamic covalent reactions^14^ for nanoparticles stabilized by ligands terminated with hydrazones,^60,62,63,66^ boronic acids,^61^ and acetals.^65^ The optimized AuNP synthesis protocol outlined above was applied to ligands bearing dynamic covalent reactive groups at their periphery to produce AuNP-**5** (solvent: THF/MeOH, 10:1 *v*/*v*; ⟨*d*⟩ = 3.10 ± 0.42 nm, 14%; Figure S19), AuNP-**6** (solvent: MeOH/DMF, 1:10 *v*/*v*; ⟨*d*⟩ = 4.87 ± 0.74 nm, 15%; Figure S22), and AuNP-**7** (solvent: THF/DMF, 9:1 *v*/*v*; ⟨*d*⟩ = 5.0 ± 0.5 nm, 10%; Figure S26).

To verify that the reactive functional groups were successfully incorporated intact within the nanoparticle-stabilizing monolayer, samples were purified from the unbound molecular species and the monolayer composition was determined by in situ and ex situ NMR spectroscopy experiments (Supporting Information, section 3). Although boranes are known to reduce amides to amines,^67,68^ we verified that the attenuated reactivity of the amine borane complex TBAB did not affect the amide functional group on AuNP-**4** (Figures S17 and S18). Likewise, oxidatively sensitive and Lewis acidic boronic acids (AuNP-**5**, Figure S21) and hydrolytically and reductively sensitive acetals (AuNP-**7**, Figures S28 and S29) were incorporated on the nanoparticle surface with no trace of side reactions. Only in the case of AuNP-**6** was any change in the molecular structure of the ligand molecule observed. When starting with the boronic acid functionalized disulfide ligand precursor **6**_2_, a small proportion (ca. 3%) of the surface-bound molecules was found to have undergone oxidative deboronation to the corresponding phenol. However, protecting the boronic acid as its pinacol ester (**S4**_2_, Scheme S1) completely suppressed this side reaction with the added benefit of spontaneous deprotection under the nanoparticle synthesis conditions, giving AuNP-**6** without requiring any further manipulations.

The moderate variation in size distribution characteristics across this series (Table 1 and Figure 6, blue data points) can likely be ascribed to solvent effects. The solvent can influence the synthetic outcome through its intrinsic role in several key parameters (see also the Controlling Nanoparticle Size by Rate of Reducing Agent Addition discussion).^46,47^ For example, we observed that including MeOH in the solvent mixture led to rapid color changes at the start of the reaction, which is indicative of a fast reduction and is consistent with smaller nanoparticle mean sizes, while the presence of DMF tended to retard reduction rates, leading to slightly larger average sizes. Nevertheless, it is clear that this single-phase, one-step protocol can be adapted to produce AuNPs stabilized by an unusually wide range of functionalized and reactive ligands when it is provided with an appropriate solvent that satisfies the solubility properties of both the ligand and the nanoparticle products. Importantly, including functionality in the ligand structure does not lead to broader size distributions; dispersities in the range of 9–17% compare favorably with those from other methods for direct preparation of gold nanoparticles.^24,31,34,44,48^

**Table 1:** Summary of the AuNP Size Distributions Obtained under Instantaneous and Slow Addition of the Reducing Agent

		⟨*d*⟩ (nm) (% dispersity)^b^		
AuNP	solvent^a^	instant addition	slow addition^c^	relative size increase
AuNP-**1**	CHCl_3_	2.71 ± 0.64 (24%)	4.84 ± 0.79 (16%)	79%
AuNP-**1**	PhMe	5.70 ± 0.51 (9%)	6.17 ± 0.77 (12%)	8%
AuNP-**2**	CHCl_3_	3.12 ± 0.72 (23%)	4.44 ± 1.19 (27%)	42%
AuNP-**2**	PhMe	7.26 ± 1.24 (17%)	9.81 ± 1.28 (13%)	35%
AuNP-**3**	THF/DMF 1:1	3.72 ± 0.58 (16%)	10.34 ± 1.28 (12%)	178%
AuNP-**4**	THF/MeOH 10:1	3.86 ± 0.39 (10%)	6.12 ± 0.66 (11%)	59%
AuNP-**5**^d^	THF/MeOH 10:1	3.10 ± 0.42(14%)	4.95 ± 0.66 (13%)	60%
AuNP-**6**	DMF/MeOH 10:1	4.28 ± 0.70 (16%)	7.18 ± 1.55 (22%)	68%
AuNP-**7**^e^	THF/DMF 9:1	4.90 ± 0.60 (10%)	8.04 ± 0.98 (12%)	64%

a Solvent mixtures expressed as volume ratios.

b Summary data: mean diameter ± standard deviation (percent dispersity). Distributions calculated by analysis of at least 100 AuNPs from multiple TEM images of the same sample. See the Supporting Information for size distribution histograms.

c Dropwise addition of TBAB, typically over a period of 2 h.

d Slow addition: TBAB added over a period of 1 h.

e Slow addition: TBAB added over a period of 0.5 h.

### Controlling Nanoparticle Size by Rate of Reducing Agent Addition

**Figure 3:**
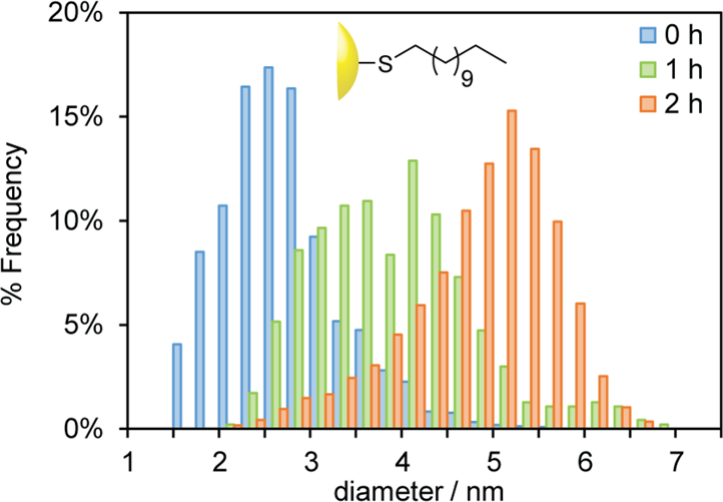
Size distributions of AuNP-**1** prepared in CHCl_3_ with the reducing agent added instantaneously (blue bars), dropwise over 1 h (green bars), or dropwise over 2 h (orange bars). Representative TEM images and size distributions in terms of the absolute count of particles can be found in Figures S1–S3.

To identify strategies for systematically tuning the AuNP core size, we reflected on our observations noted above and on those of others^49^ that the conditions under which the metal precursor reduction occurred the fastest tended to give smaller particles, and vice versa. Therefore, we repeated the synthesis of dodecanethiyl-stabilized AuNP-**1** in CHCl_3_ as before, but now added the reducing agent slowly via a syringe pump over the course of 2 h. Under these conditions, a significantly larger population of AuNP-**1** with ⟨*d*⟩ = 4.84 ± 0.79 nm (16% dispersity; Figures 2 and S3) was obtained, corresponding to a 79% increase in mean diameter and accompanied by an improvement in size dispersity (Figure 3, orange bars). This outcome was highly reproducible. Over three batches, the same protocol consistently yielded monomodal particle distributions with mean diameters in the range of 4.57–4.84 nm and dispersities of 12–18% (Figures S3–S5). Shortening the time over which TBAB was added to 1 h resulted in an intermediate distribution of sizes between that of the instantaneous addition and that of the 2 h addition populations (⟨*d*⟩ = 3.90 ± 0.86 nm; Figure 3, green bars, and Figure S2). While this was an encouraging result, even larger dodecanethiol-coated AuNPs could already be accessed by changing the synthesis solvent from CHCl_3_ to toluene (vide supra). Nonetheless, the majority of the more structurally sophisticated ligands do not allow for such wide flexibility in the synthesis solvent; therefore, we were intrigued by the prospect of a method for varying AuNP size that might prove to be systematic and general across a wide range of ligand types.

We reasoned that a similar increase in size to give AuNP-**1** with ⟨*d*⟩ > 6 nm might be achieved by slowly adding the reducing agent to the synthesis of AuNP-**1** in toluene. Disappointingly, adding TBAB to the reaction in toluene over a period of 2 h produced AuNP-**1** with an average diameter of 6.17 ± 0.77 nm, almost identical to the sample obtained after instantaneous addition of the reducing agent (5.70 ± 0.51 nm, compare Figures S6 and S7). Visual inspection of the reaction mixture indicated that AuNP-**1** of this size is not well solubilized in toluene, suggesting that for each ligand/solvent pair there may be an intrinsic size limit beyond which precipitation removes nanoparticles from the growth process. In an attempt to stabilize larger nanoparticles in toluene, we switched to the longer octadecanethiol ligand precursor. In this case, instant addition of the reducing agent in toluene already afforded AuNP-**2** with a size of ⟨*d*⟩ = 7.3 nm (vide supra); pleasingly, adding the reducing agent slowly over a period of 2 h resulted in a further increase in mean size of ca. 35% to give AuNP-**2** with ⟨*d*⟩ = 9.8 nm, again with no broadening in the size distribution (Figures 6 and S11). Likewise, when using CHCl_3_ as the reaction solvent for preparing AuNP-**2**, a size increase of ca. 40% was observed with slow addition of the reducing agent, giving ⟨*d*⟩ = 4.4 nm (Figures 6 and S9). This result was encouraging, as it suggested that decreasing the rate of addition in order to increase the nanoparticle size is a general principle, independent of the specific solvent or ligand employed.

**Figure 4:**
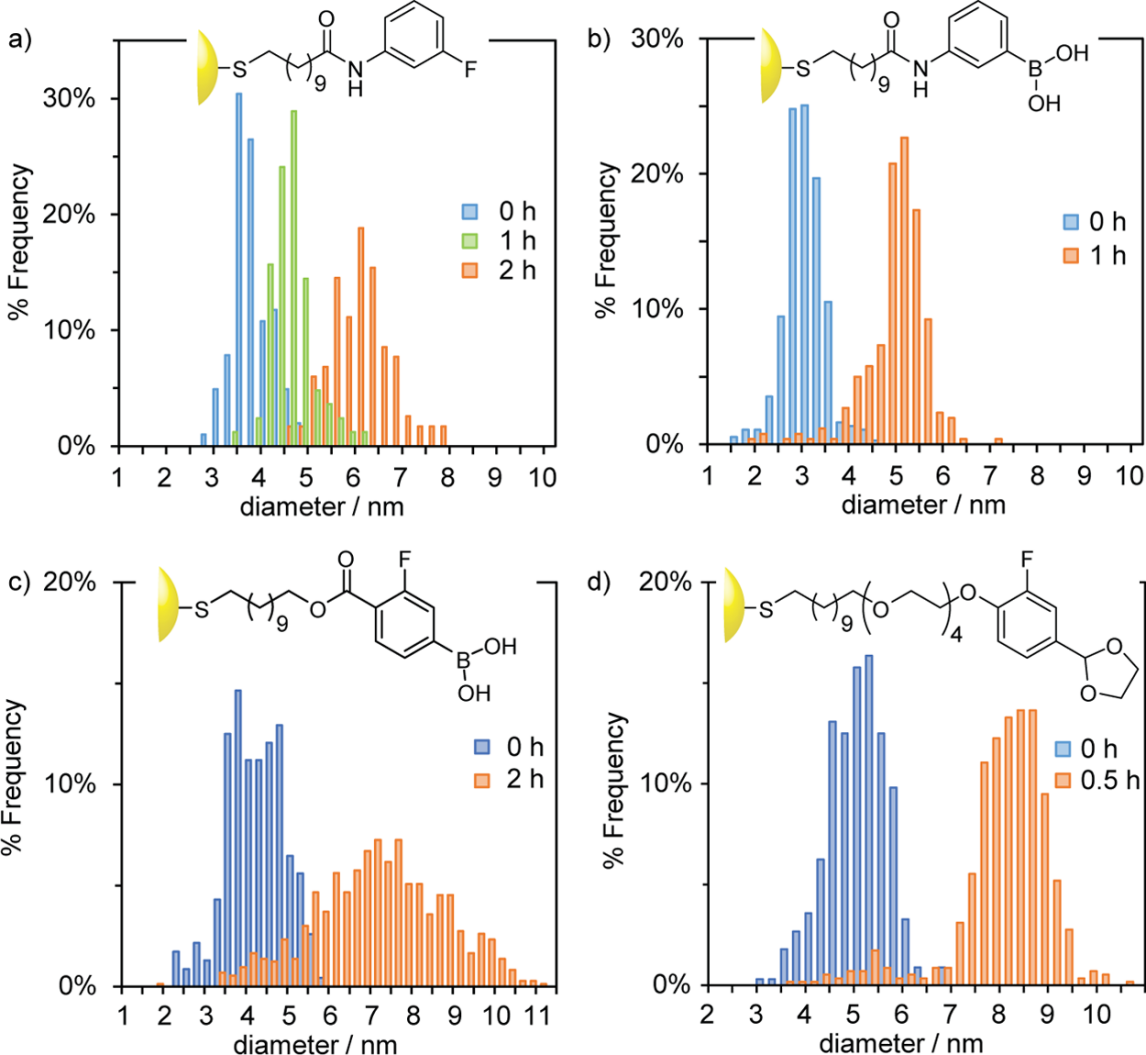
Size distributions of (a) AuNP-**4** prepared in THF/MeOH (10:1 *v*/*v*) with the reducing agent added instantaneously (blue bars), dropwise over 1 h (green bars), or dropwise over 2 h (orange bars); (b) AuNP-**5** prepared in THF/MeOH (10:1 *v*/*v*) with the reducing agent added instantaneously (blue bars) or dropwise over 1 h (orange bars); (c) AuNP-**6** prepared in DMF/MeOH (10:1 *v*/*v*) with the reducing agent added instantaneously (blue bars) or dropwise over 2 h (orange bars); and (d) AuNP-**7** prepared in THF/DMF (9:1 *v*/*v*) with the reducing agent added instantaneously (blue bars) or dropwise over 0.5 h (orange bars). Representative TEM images and size distributions in terms of the absolute count of particles can be found in Figures S14–S16 (AuNP-**4**), S19 and S20 (AuNP-**5**), S22 and S23 (AuNP-**6**), and S26 and S27 (AuNP-**7**).

**Figure 5:**
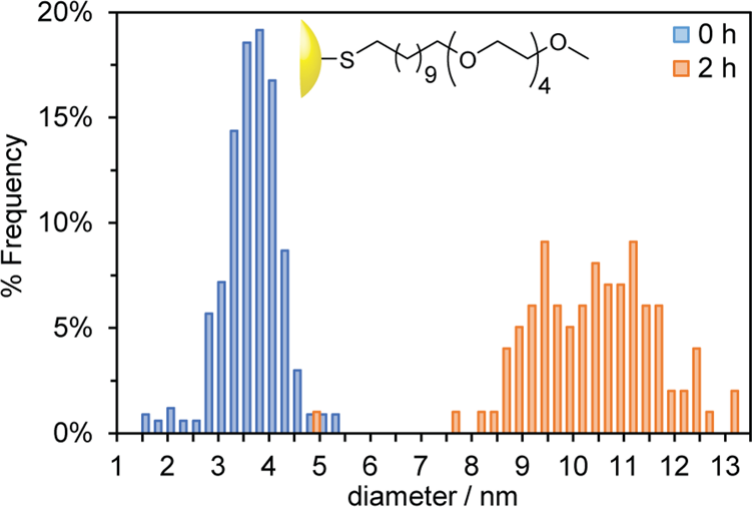
Size distributions of AuNP-**3** prepared in DMF/THF (1:1 *v*/*v*) with the reducing agent added instantaneously (blue bars) or dropwise over 2 h (orange bars). Representative TEM images and size distributions in terms of the absolute counts of particles can be found in Figures S12 and S13.

**Figure 6:**
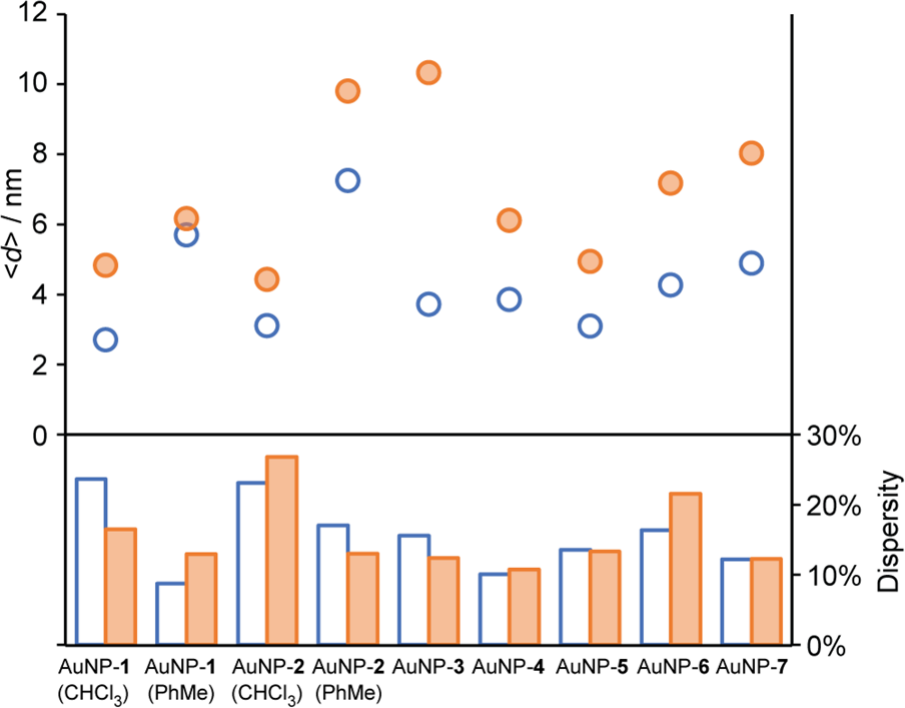
A summary of nanoparticle size distributions expressed in terms of mean diameter (top panel) and relative dispersity (bottom panel) achieved by the instantaneous addition of the reducing agent (blue open symbols) and the slow addition of the reducing agent (orange shaded symbols). See Table 1 for a summary of the particle size distribution statistics and the Supporting Information for distribution histograms and representative TEM images.

We repeated the syntheses of each of AuNP-**3**–AuNP-**7**, adding the reducing agent slowly, with the results summarized in Figures 4–6 and in Table 1. In all cases, a significant (>50%) increase in the mean diameter was observed when the reductant was added slowly. For AuNP-**4**, a 60% increase in the mean size was observed on addition of TBAB over a period of 2 h, giving ⟨*d*⟩ = 6.12 ± 0.66 nm (Figures 4a and S16). As before, adding the reductant over an intermediate time period of 1 h produced a particle distribution intermediate in size (⟨*d*⟩ = 4.30 ± 0.39 nm; Figures 4a and S15) between the instant and 2 h addition conditions. For boronic acid-stabilized AuNP-**5**, adding the reductant over the course of 2 h resulted in precipitation of a highly aggregated product, rendering quantitative sizing by TEM imaging impossible. However, images where smaller aggregates could be seen suggested that the nanoparticles were approximately 6 nm in diameter. Assuming that aggregation had resulted from the nanoparticles growing beyond the intrinsic limit for this ligand in the synthesis solvent, we shortened the period over which the reducing agent was added, providing a stable solution of AuNP-**5** with ⟨*d*⟩ = 4.95 ± 0.66 nm (Figures 4b and S20)—an increase of 60%. Likewise, for AuNP-**7**, slow reductant addition over a period of 2 h produced insoluble material in THF/DMF (9:1 *v*/*v*). In this case, adding TBAB over only the course of 0.5 h was sufficient to generate a 64% increase in the mean diameter, giving fully soluble AuNP-**7** with ⟨*d*⟩ = 8.04 ± 0.99 nm (Figures 4d and S27). The most striking result was observed for tetra(ethylene glycol)-stabilized AuNP-**3**, for which slow addition of the reducing agent over a period of 2 h resulted in a 178% increase in mean diameter to 10.3 ± 1.3 nm (Figures 5 and S13), again with a negligible effect on the relative size dispersity (Figure 6). This result is consistent with the significantly longer ligand being able to stabilize much larger nanoparticles in the optimized reaction solvent.

Solution-phase nanoparticle synthesis requires two events: nucleation and growth.^69−71^ Endothermic nucleation involves bond cleavage in the precursor, removal of the solvate shells, and (in the case of metallic cores) precursor reduction to the elemental oxidation state. This is followed by exothermic growth, driven by the bulk solid lattice enthalpy and the minimization of high-energy surface sites. Stabilizers are thus critical for modulating growth in order to produce colloidally stable nanoparticles. For the reduction of transition metals, nucleation and growth phases typically temporally overlap. Several processes can contribute during the growth phase, including diffusion-limited monomer attachment, autocatalytic metal cation reduction at the nanoparticle surface, aggregative growth, and Ostwald ripening.^69,70^ Although the kinetic mechanisms for particle formation and growth under the conditions introduced by Stucky have not been elucidated in detail, it is proposed that nucleation produces small, phosphine-stabilized clusters.^72,73^ Strongly aurophilic thiols or thiolates etch less stable clusters,^28,74,75^ producing a reservoir of neutral thiolate–gold complexes,^74−76^ which in turn displace neutral phosphine ligands on the more stable clusters, leading to nanoparticle growth concurrent with the assembly of the stabilizing alkanethiyl monolayer.^47^ The solvent and solvation characteristics of the stabilizing ligand(s) will therefore be critical determinants of the nanoparticle size distribution through their influence on several mechanistically important parameters. These include (1) the reactivity of the borane reducing agent and, hence, the duration and overlap of the nucleation and growth phases; (2) the supersaturation concentration of Au(0) required for nucleation and, hence, the concentration of the nuclei generated; (3) the surface free energy of the nuclei and the ligand donor atom nucleophilicity, which affect the etching rate of the phosphine-stabilized clusters; (4) the saturation concentration of the reservoir of Au–thiolate monomers for particle growth; and (5) the solubility/colloidal stability of growing the nanoparticle products. This mechanistic complexity underlines the importance, and challenge, of empirically optimizing solvent mixtures for each stabilizing ligand and explains the variation in nanoparticle size distribution observed according to the surface ligand structure and solvent. We found that decreasing the rate of reductant addition beyond 2 h failed to reproducibly generate even larger particles, suggesting that an intrinsic limit had been reached for the examples investigated. Nevertheless, we expect that optimizing the solvent characteristics to match the characteristics of each ligand could generate larger particles.

In light of the mechanistic complexity, it is remarkable that a simple control parameter, the rate of the reducing agent addition, has a consistent effect on nanoparticle size irrespective of the ligand structural characteristics, without also harming dispersity (Figure 6). Under Brust–Schiffrin conditions, the rate of borohydride addition has been observed to influence nanoparticle size distribution, but only when varied in conjunction with changes to the stoichiometry of the stabilizer.^45^ By contrast, we have shown that the rate of addition of borane to a Au(I) precursor can act as an independent control parameter. The characteristics of the final nanoparticle population are encoded in the nucleation process.^70^ Adding the reducing agent slowly means that fewer nuclei are produced in the initial stages. Subsequently, the concentration of Au(0) atoms is limited by slow reduction and rapid consumption in the nanoparticle growth processes. Even if there is some overlap of the nucleation and growth phases, this means that the slow addition of the reducing agent does not extend the nucleation phase, which would broaden the size distribution. Instead, slow gold reduction in the presence of an excess of thiols maintains a reservoir of the Au–thiolate monomer species, leading to continued growth of pre-existing particles without nucleating new particles. Overall, the system can be viewed as an in situ seeded growth mechanism. This picture is consistent with the observation that size dispersity is in many cases slightly improved by the slow addition of the reducing agent (Figure 6) and that particle size can be tuned within the limits identified in Table 1 by adjusting the rate of addition (e.g., Figures 3 and 4a).

A similar seeded growth mechanism has been elucidated from in situ studies on the formation of citrate-stabilized AuNPs using a water-soluble Au(III) precursor under the classic Turkevich–Frens conditions.^77^ Rapid initial nucleation that consumes only a fraction of the gold precursor is followed by an extended diffusional growth and size-focusing phase, which is controlled by the slow kinetics for further Au(III) reduction. Although TBAB is a stronger reducing agent than citrate, adding TBAB slowly over an extended period can be expected to create similar conditions for slow nanoparticle growth by monomer attachment while still maintaining monomer concentrations below the critical nucleation concentration, thus leading to the observed increase in particle size without affecting dispersity.^78^

## Conclusions

In summary, we have identified a one-step direct synthesis protocol for generating gold nanoparticles stabilized by a wide variety of ligand structures with systematic control over the nanoparticle size via a single reaction parameter. The single-phase reduction of an organic-soluble Au(I) complex using a mild borane reducing agent under gentle heating is compatible with a variety of ligand structures and functional groups, and the nanoparticle size distributions are not broadened by introducing functionality or structural complexity in the surface-stabilizing ligand.

The critical factor that determines successful nanoparticle synthesis is identifying a reaction solvent that solubilizes all of the starting materials, as well as nascent nanoparticles during the growth process. Correspondingly, the ligand structure can be designed to produce nanoparticles with vastly differing solubility properties, or the solvent can be chosen to match the properties of a given desired ligand without changing the synthesis procedure. Although some solvent-dependent variation in nanoparticle size is observed by us and others,^46,47^ the solvent type does not provide a general strategy for size tuning because its influence is unpredictable and severely limited by the narrow, structure-dependent solvent compatibility of functionally sophisticated ligands. By contrast, the rate of reducing agent addition is an independent parameter that has a consistent effect on nanoparticle size: slower rates of addition lead to larger nanoparticles, importantly without harming size dispersity. We found that the limit of nanoparticle growth is set by the characteristics of each specific ligand/solvent pairing. Irrespective of the reduction rate, the relative dispersities of the nanoparticle size distributions compare favorably with those of other direct synthesis protocols for gold nanoparticles stabilized by functionalized ligands.

This method has a number of distinctive advantages. The one-step procedure is operationally straightforward; it is time- and material-efficient and can be used to produce nanoparticles with widely differing properties without requiring lengthy case-by-case optimization. Single-phase reactions avoid inhomogeneities that can arise from stochastic interfacial events in biphasic systems and do not require phase-transfer agents. No temporary surface-stabilizing additives are required only the weakly binding phosphines that come from the precursor gold complex. Consequently, the only species present that can bind strongly to the gold surface are the chosen sulfur-based ligand(s), predictably generating single-component self-assembled monolayers. This has several attractive implications for maximizing the influence of a given ligand design on nanoparticle physicochemical properties,^10,60,65^ enabling in situ molecular-level characterization of surface-bound functionalities,^60,63,66^ and minimizing batch-to-batch variability that may result from monolayer impurities. In contrast to many ligand-exchange protocols, the synthetically costly functional ligand is used in relatively modest excess, and the number of manipulations is minimized, making for more efficient and convenient procedures. Spectroscopic analysis of the nanoparticle products has verified that a range of functional group types in the surface-stabilizing ligands is compatible with this method, allowing additive properties such as conjugation sites or analytical handles to be introduced in a single-step nanoparticle preparation procedure.

As well as allowing for the efficient construction of functionally sophisticated nanoparticles, this protocol meets several of the critical requirements for producing chemically reactive colloidal nanoparticle “building blocks”^14,61,63,66^ while still achieving systematic control over core size. Independent control over structural features of the core and the surface-stabilizing monolayer is necessary in order to access the full range of nanoparticle properties, while the ability to include reactive molecular functionality in the surface-bound ligands is essential for enabling divergent routes to a variety of functional nanoparticle-based devices and materials. Altogether, predictable synthetic strategies of this nature will accelerate efforts to explore and exploit nanoscale chemical space.

## Data Availability

Underpinning data available via University of St Andrews Research Portal: https://doi.org/10.17630/385fe69b-6192-4604-8c1f-ba68e14b404f.

## Supporting Information Available

General experimental details, procedures for preparation of the ligand precursors, supplementary TEM images and nanoparticle size distributions for all experiments and replicates, and in situ and ex situ ^1^H and ^19^F NMR spectra for functionalized ligand nanoparticles (AuNP-**4**–AuNP-**7**) (PDF)
