# Supplementary material for: A General One-Step Synthesis of Alkanethiyl-Stabilized Gold Nanoparticles with Control over Core Size and Monolayer Functionality
Source: Chem Mater. 2023 Jul 17;35(15):6168–77. doi: 10.1021/acs.chemmater.3c01506 (PMC10413864; doi:10.1021/acs.chemmater.3c01506)
Supplement: Supplementary file 1 — cm3c01506_si_001.pdf [file cm3c01506_si_001.pdf]

Supporting Information

A General One-Step Synthesis of Alkanethiyl-Stabilized Gold Nanoparticles with Control over Core Size and Monolayer Functionality

Stefan Borsley, William Edwards, Ioulia K. Mati, Guillaume Poss, Marta Diez-Castellnou, Nicolas Marro, Euan R. Kay*

1. General experimental procedures S2
2. Synthesis of molecular species S3
3. Nanoparticle synthesis and characterization S5
4. References S26

1. General experimental procedures

Unless stated otherwise, all reagents were purchased from commercial sources and used without further purification. Prior to use, traces of dimethylamine were removed from DMF by evaporation under vacuum with gentle heating, followed by cooling to room temperature under vacuum then storage under a nitrogen atmosphere. All other solvents were reagent grade and used as received. Flash column chromatography was performed using Geduran® Si60 (40–63 μm, Merck, Germany) as the stationary phase. Thin-layer chromatography (TLC) was performed on pre-coated silica gel plates (0.25 mm thick, $60F_{254}$, Merck, Germany) and observed under UV light (λ_{max} 254 nm) or visualized by staining with a basic potassium permanganate solution, followed by heating. Nanoparticle micrographs were obtained using a JEM 2010 transmission electron microscope (TEM) on samples prepared by deposition of one drop of nanoparticle suspension on holey carbon films supported on a 300 mesh Cu grid (Agar Scientific®). Nanoparticle diameters were measured automatically using the software *ImageJ*. The images were first converted to black and white images using the "Threshold" function; the area of each nanoparticle was measured using the "Analyze particles" function; particles on edges were excluded. ^{1}H, ^{13}C, and ^{19}F NMR spectra were recorded on Bruker AV 400, AV-II 400 and AVIII-HD 500 instruments, at a constant temperature of 25 °C. ^{1}H and ^{13}C chemical shifts are reported in parts per million (ppm) from low to high field and referenced to the literature values for chemical shifts of residual non-deuterated solvent, with respect to tetramethylsilane. ^{19}F Chemical shifts are referenced to CF_3Cl (0.00 ppm) as external standard. Standard abbreviations indicating multiplicity are used as follows: bs (broad singlet), d (doublet), dd (doublet of doublets), dt (doublet of triplets), m (multiplet), s (singlet), t (triplet), tt (triplet of triplets), q (quartet), quint (quintuplet), *J* (coupling constant). All spectra were analyzed using MestReNova (Versions 9.0.0–12.0.02). Mass spectrometry was carried out on a Waters GCT Premier or a LTQ Orbitrap XL at the EPSRC UK National Mass Spectrometry Facility at Swansea University or on a Thermo Exactive Orbitrap mass spectrometer at the University of St Andrews. A Beckman Coulter Avanti J-25 centrifuge was used equipped with the JA-25.50 rotor.

2. Synthesis of molecular species

Chloro(triphenylphosphine)gold(I),[1] **S1**,[1] **S3**$_2$,[2] **S4**$_2$,[3] **5**$_2$,[2] **7**$_2$,[4] were all prepared as previously described. Compounds **3**$_2$ and **4**$_2$ were prepared according to the synthetic sequence shown in Scheme S1.

Note that the same compound number is used to indicate a given alkylsulfanyl structure in molecular and nanoparticle-bound formats using the following convention: **x**H = thiol, **x**$_2$ = disulfide, AuNP-**x** = nanoparticle-bound.

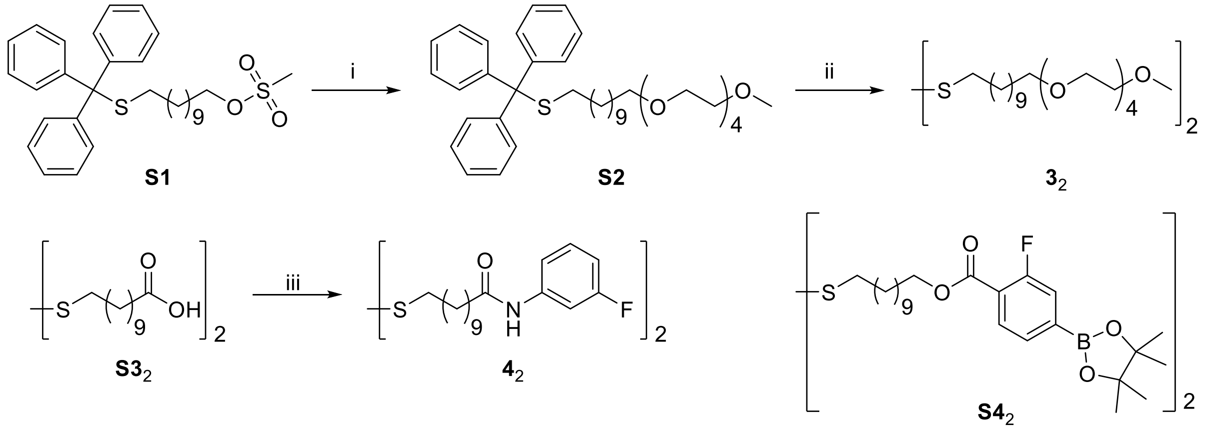

Scheme S1. Synthesis of pro-ligands **3**$_2$ and **4**$_2$ and the molecular structure of **S4**$_2$. Reagents and conditions: (i) Tetraethylene glycol monomethyl ether, NaOH, H_2O, 90 °C, overnight, 22%. (ii) I_2, MeOH, CH_2Cl_2, r.t., 2 h, 97%. (iii) 3-Methoxyaniline, EDC•HCl, HOBt, *N*,*N*-diisopropylethylamine, MeCN/THF (1:2 *v*/*v*), r.t., 18 h, 77%.

(3-(2-Methoxyethoxy)propyl)(trityl)sulfane (S2)

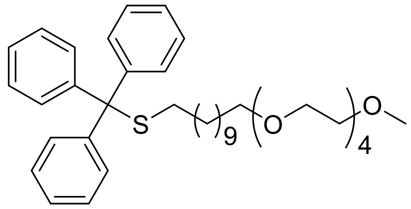

A solution of NaOH (183 mg, 4.57 mmol) in H_2O (200 μL) was added to tetraethylene glycol monomethyl ether (1.14 mL, 5.72 mmol) in a 25mL round-bottom flask equipped with a reflux condenser. The mixture was heated to 90 °C for 1 h. Then, compound **S1** (ref. [1]) (2.00 g, 3.81 mmol) was added and heating continued at 90 °C for 16 h. The reaction mixture was allowed to cool to room temperature and then was poured into diethyl ether (100 mL). The organic phase was washed with saturated aqueous $NaHCO_3$ (sat. aq., 2 × 50 mL) and brine (2 x 50 mL). The organic layer was dried over $MgSO_4$, filtered and solvent was removed under reduced pressure to obtain a yellowish oil. The crude product was purified by flash column chromatography (SiO_2, petroleum ether/EtOAc 4:1 to 1:1) to obtain **S2** as a colorless oil (545 mg, yield: 22%). ^{1}H NMR (500.1 MHz, $CDCl_3$): δ = 7.43–7.39 (m, 6H, Ar*H*), 7.25–7.29 (m, 6H, Ar*H*), 7.20 (tt, *J* = 2.1 Hz, *J* = 7.3 Hz, 3H, Ar*H*), 3.62–3.67 (m, 12H, 6 × C*H*$_2$O), 3.53–3.59 (m, 4H, 2 × C*H*$_2$O), 3.44 (t, *J* = 6.8 Hz, 2H, C*H*$_2$O), 3.38 (s, 3H, OC*H*$_3$), 2.12 (t, *J* = 7.4 Hz, 2H, C*H*$_2$S), 1.56 (quint, *J* = 7.7 Hz, 2H, C*H*$_2$), 1.38 (quint, *J* = 7.6 Hz, 2H, C*H*$_2$), 1.10–1.30 (m, 14H, 7 × CH_2); ^{13}C{^{1}H} NMR (125.8 MHz, $CDCl_3$): δ = 145.2, 129.7, 127.9, 126.6, 72.1, 71.7, 70.7, 70.7, 70.6, 70.2, 66.5, 59.2, 32.2, 29.8, 29.7, 29.6, 29.6, 29.5, 29.3, 29.1, 28.7, 26.2; HRMS (ESI$^+$) m/z calculated for $[M+Na]^+$ $C_{39}H_{56}NaO_5S$ 659.3741, found 659.3724.

2,5,14,17-Tetraoxa-9,10-dithiaoctadecane (3$_2$)

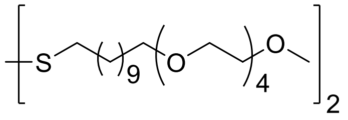

Compound **S2** (330 mg, 0.52 mmol) was dissolved in a mixture of MeOH/CH_2Cl_2 (3:1 *v*/*v*, 20 mL) in a round-bottomed flask. Iodine (114 mg, 0.57 mmol) was then added and the mixture was stirred at

room temperature. The reaction was monitored using TLC (4% MeOH/CH_2Cl_2), and as soon as the starting material was consumed (ca. 2 h after adding iodine), the reaction was stopped immediately by removing the excess of iodine by washing with a saturated aqueous solution of $NaHSO_3$ (50 mL). The aqueous layer was extracted with CH_2Cl_2 (3 × 50 mL). The organic layers were then combined, washed with brine (2 × 50 mL), dried over $MgSO_4$ and solvent was removed under reduced pressure. The crude product was purified by flash column chromatography (SiO_2, 4% MeOH/CH_2Cl_2) to obtain **3**$_2$ as a colorless oil (204 mg, yield: 97%). ^{1}H NMR (500.1 MHz, $CDCl_3$): δ = 3.62–3.68 (m, 24H, 12 × CH_2O), 3.53–3.59 (m, 8H, 4 × CH_2O), 3.44 (t, *J* = 6.9 Hz, 4H, 2 × CH_2O), 3.38 (s, 6H, 2 × OCH_3), 2.67 (t, *J* = 7.5 Hz, 4H, 2 × CH_2S), 1.66 (quint, *J* = 7.5 Hz, 4H, 2 × CH_2), 1.57 (quint, *J* = 7.3 Hz, 4H, 2 × CH_2), 1.23–1.40 (m, 28H, 14 × CH_2); ^{13}C{^{1}H} NMR (125.8 MHz, $CDCl_3$): δ = 72.1, 71.7, 70.7, 70.7, 70.7, 70.2, 59.2, 39.3, 29.8, 29.7, 29.7, 29.6, 29.6, 29.4, 29.4, 28.7, 26.2; HRMS (ESI$^+$) m/z calculated for $[M+H]^+$ $C_{40}H_{82}O_{10}S_2$ 787.5422, found 787.5406.

11,11'-Disulfanediylbis(*N*-(3-fluorophenyl)undecanamide) (**4**$_2$)

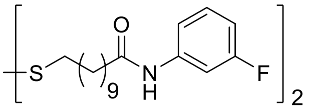

11,11'-Disulfanediyldiundecanoic acid (**S3**$_2$) (ref. [2]) (1.00 g, 2.30 mmol), 3-fluoroaniline (0.853 g, 5.50 mmol) and EDC•HCl (1.10 g, 5.75 mmol) were dissolved in THF (10 mL) and MeCN (5 mL). *N*,*N*-Diisopropylethylamine (1.49 g, 11.5 mmol) was added and the reaction was stirred at room temperature for 16 h. The solution was poured into a mixture of EtOAc and 1 M HCl. The organic layer was washed with 1 M HCl, $NaHCO_3$ (sat. aq.) and brine, then dried over $MgSO_4$. Solvent was removed under reduced pressure to give a pale yellow solid. This solid was sonicated in MeOH and filtered to give the desired product **4**$_2$ as a white solid. (1.26 g, 2.03 mmol, 88%); ^{1}H NMR (500.1 MHz; $CDCl_3$): δ = 8.93 (2H, br s, N*H*), 7.39 (2H, d, *J* = 11.5, Ar*H*), 7.17–7.11 (4H, m, Ar*H*), 6.71–6.64 (2H, m, Ar*H*), *2*.59 (4H, t, *J* = 7.5, CH_2S), 2.25 (4H, t, *J* = 7.5, CH_2CO) 1.63–1.52 (8H, m, CH_2), 1.34–1.16 (24H, m, CH_2); ^{13}C{^{1}H} NMR (125.8 MHz; $CDCl_3$): δ = 178.2 (*C*), 172.9–170.9 (d, *J* = 252 Hz, *C*F), 139.1 (*C*), 129.9–129.8 (d, *J* = 13 Hz, *C*H), 115.0 (d, *J* = 2 Hz, *C*H), 110.5–110.4 (d, *J* = 21 Hz, *C*H), 107.2–107.0 (d, *J* = 25 Hz, *C*H), 39.1 (*C*H_2), 39.1 (*C*H_2), 29.4 (*C*H_2), 29.4 (*C*H_2), 29.4 (*C*H_2), 29.3 (*C*H_2), 29.2 (*C*H_2), 29.1 (*C*H_2), 28.4 (*C*H_2), 25.6 (*C*H_2); ^{19}F{^{1}H} NMR (470.4 MHz, $CDCl_3$): δ = –108.67; HRMS (ES$^+$) *m/z* calculated for $[M+H]^+$ $C_{34}H_{51}F_2N_2O_2S_2^+$ 612.3355, found 612.3345.

3. Nanoparticle synthesis and characterization

General nanoparticle synthetic procedure: instant addition of reducing agent

Reaction scale was typically 20–25 mg (40–50 μmol) of gold precursor, for which a 10 mL 2-neck round-bottom flask was used. $AuPPh_3Cl$ (1 mol. equiv.) and ligand precursor (thiol or disulfide, 1.2 mol. equiv. in terms of sulfur) were dissolved in the reaction solvent to give [Au] ≈ 16.7 mM. The mixture was heated to 55 °C while stirring vigorously. Borane *tert*-butylamine complex (10 mol. equiv.) in the reaction solvent (0.5 M) was then added rapidly by syringe to give a final concentration of [Au] ≈ 16 mM. Stirring was continued at 55 °C for 2 h and then at room temperature for a further 16 h.

General nanoparticle synthetic procedure: slow addition of reducing agent

Reaction scale was typically 20–25 mg (40–50 μmol) of gold precursor, for which a 10 mL 2-neck round-bottom flask was used. $AuPPh_3Cl$ (1 mol. equiv.) and ligand precursor (thiol or disulfide, 1.2 mol. equiv. in terms of sulfur) were dissolved in the reaction solvent to give [Au] ≈ 16.7 mM. The mixture was heated to 55 °C while stirring vigorously. Borane *tert*-butylamine complex (10 mol. equiv.), in the reaction solvent (0.5 M) was then added using a syringe pump at a controlled rate to achieve total addition over the intended time period. Heating was continued at 55 °C for a total of 2 h (including the time taken for addition of reducing agent), then heating was removed and stirring continued at room temperature for a further 16 h.

Nanoparticle isolation and characterization

Nanoparticles were isolated by ensuring complete precipitation with a non-solvent. When necessary, precipitation was aided by centrifugation (1312 ×g rcf, 5 °C, 10 min). The colorless supernatant was carefully decanted. The solid residue was resuspended in a good solvent (typically the reaction solvent) with sonication to ensure any material adhering to the vessel walls was redispersed prior to analysis by TEM. Consequently, the reported size distributions reflect only the outcome of the synthesis process, independent of any alteration of size distributions that might be possible via optimized purification methods such as size-selective precipitation or size-exclusion chromatography.

Prior to the assessment of ligand molecular structure by NMR spectroscopy, nanoparticles were rigorously purified from unbound molecular species by washing the black solid was with a non-solvent (5 times) with the aid of sonication. On each cycle, the supernatant was removed carefully after centrifugation (1312 ×g rcf, 5 °C, 10 min). After the final cycle, traces of solvent were removed under reduced pressure to give a black solid.

In situ NMR spectra were acquired after dispersing the nanoparticles in an appropriate deuterated solvent.

To characterize surface-bound components in solution, the nanoparticle dispersion in deuterated solvent was treated with iodine (2 mg), then the ^{1}H and ^{19}F NMR spectra recorded immediately.

AuNP-1

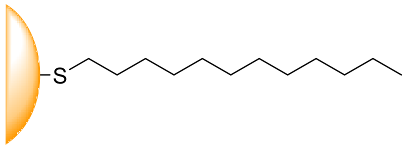

Ligand precursor: dodecanethiol (**1**)

Reaction solvent: $CHCl_3$ or toluene

Non-solvent for nanoparticle precipitation: EtOH

Slow addition time period: 1 h or 2 h.

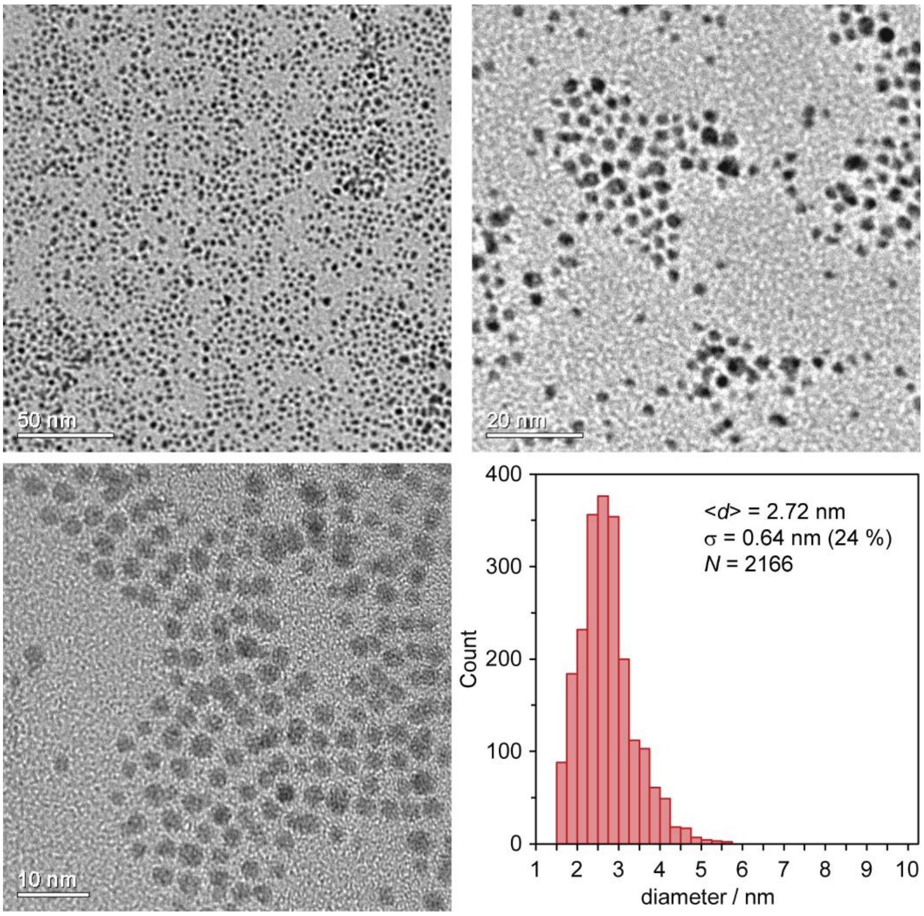

Figure S1. AuNP-**1**, $CHCl_3$, instant addition. Representative TEM images and size histogram.

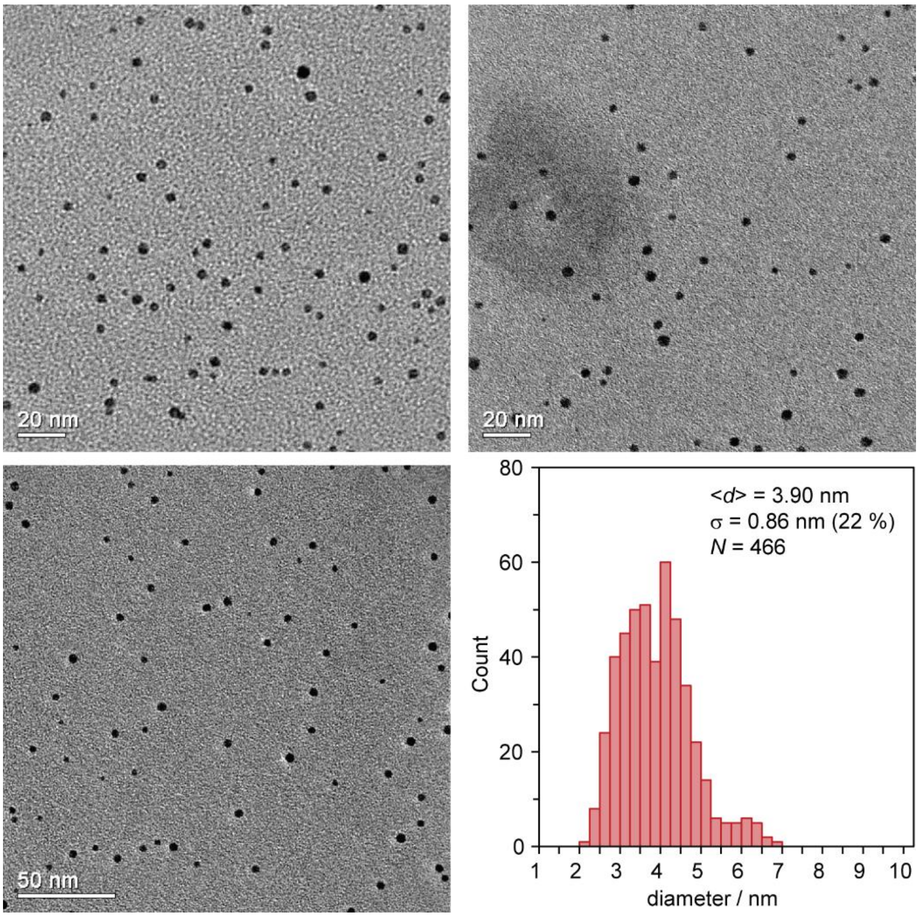

Figure S2. AuNP-**1**, $CHCl_3$, slow addition over 1 h. Representative TEM images and size histogram.

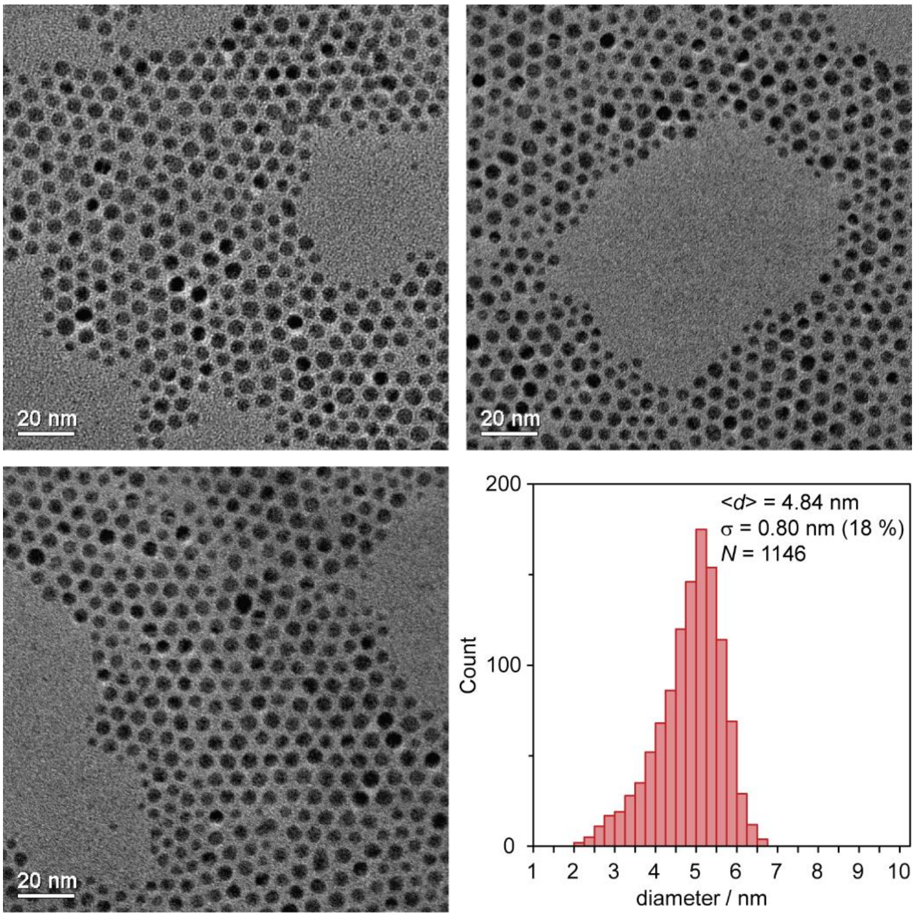

Figure S3. AuNP-**1**, $CHCl_3$, slow addition over 2 h. Representative TEM images and size histogram.

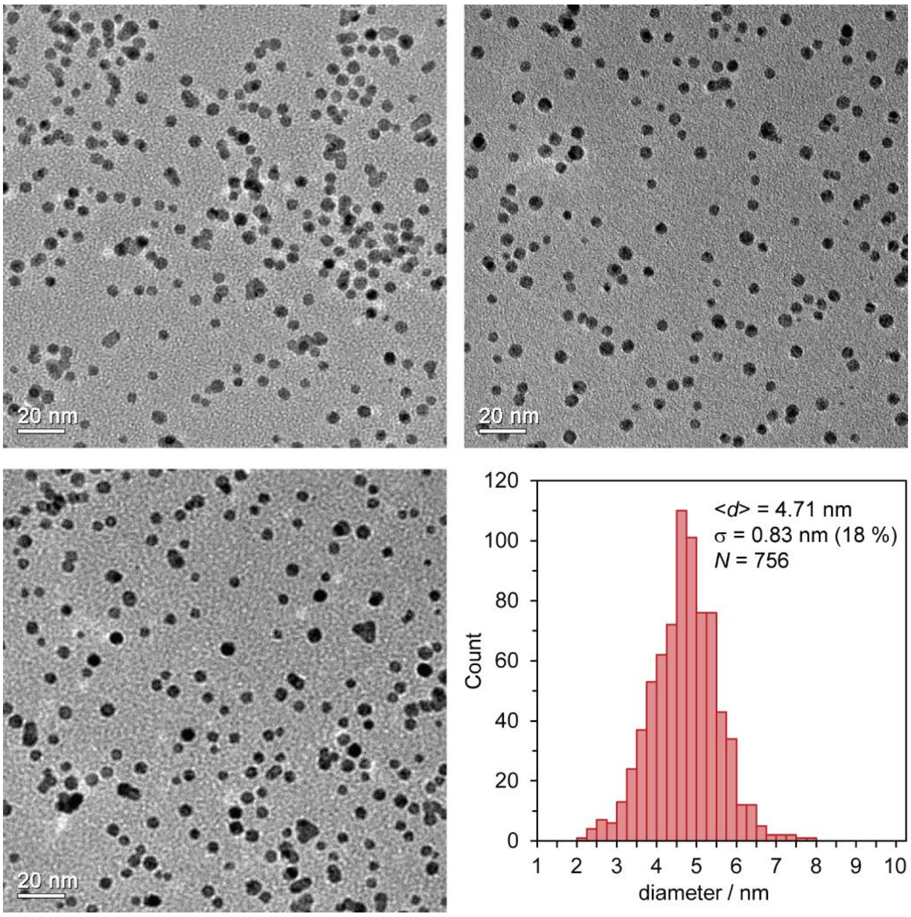

Figure S4. AuNP-**1**, $CHCl_3$, slow addition over 2 h, replicate A. Representative TEM images and size histogram.

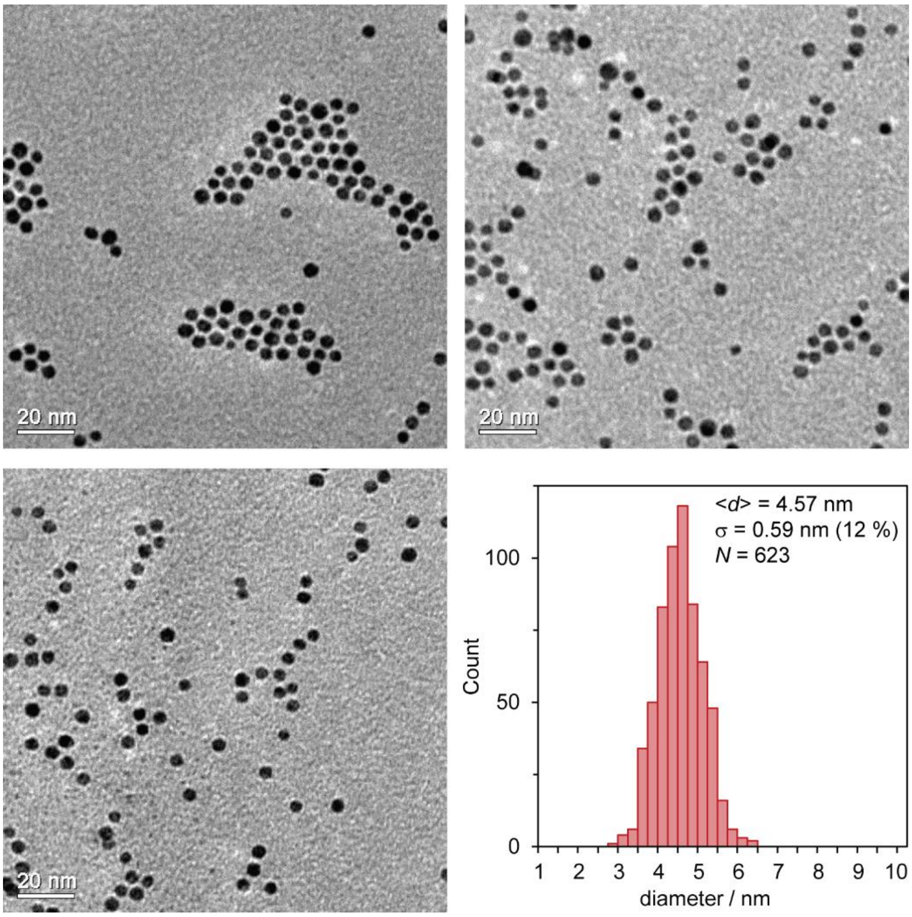

Figure S5. AuNP-**1**, $CHCl_3$, slow addition over 2 h, replicate B. Representative TEM images and size histogram.

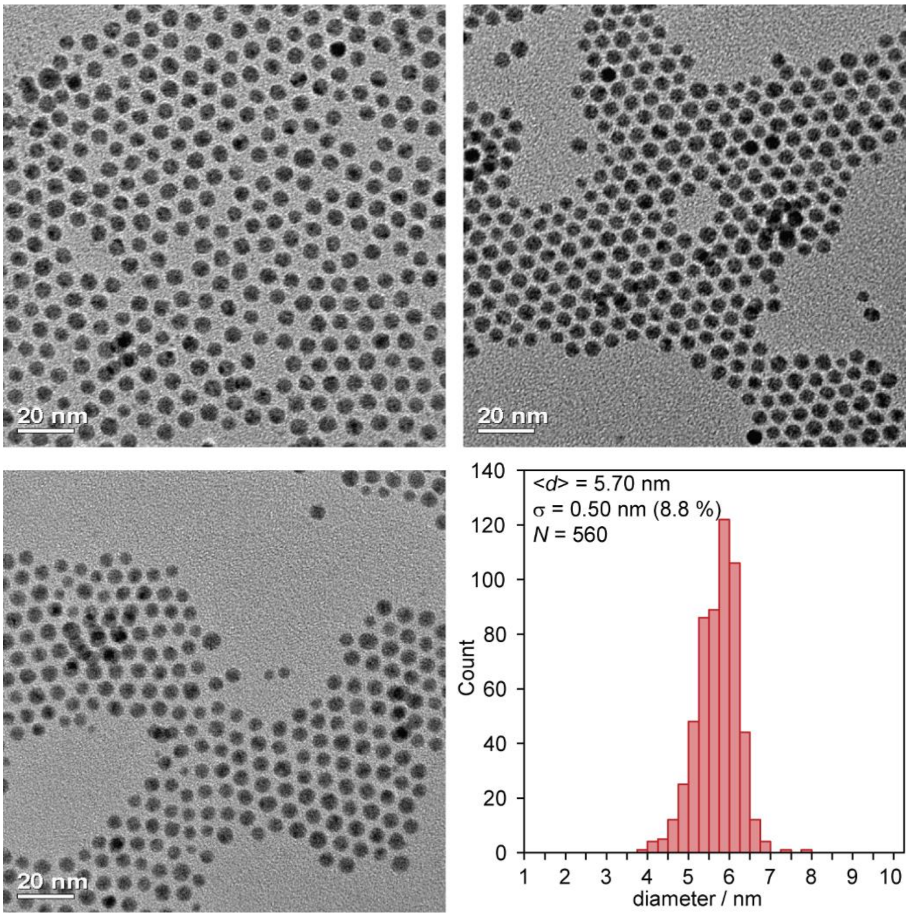

Figure S6. AuNP-**1**, toluene, instant addition. Representative TEM images and size histogram.

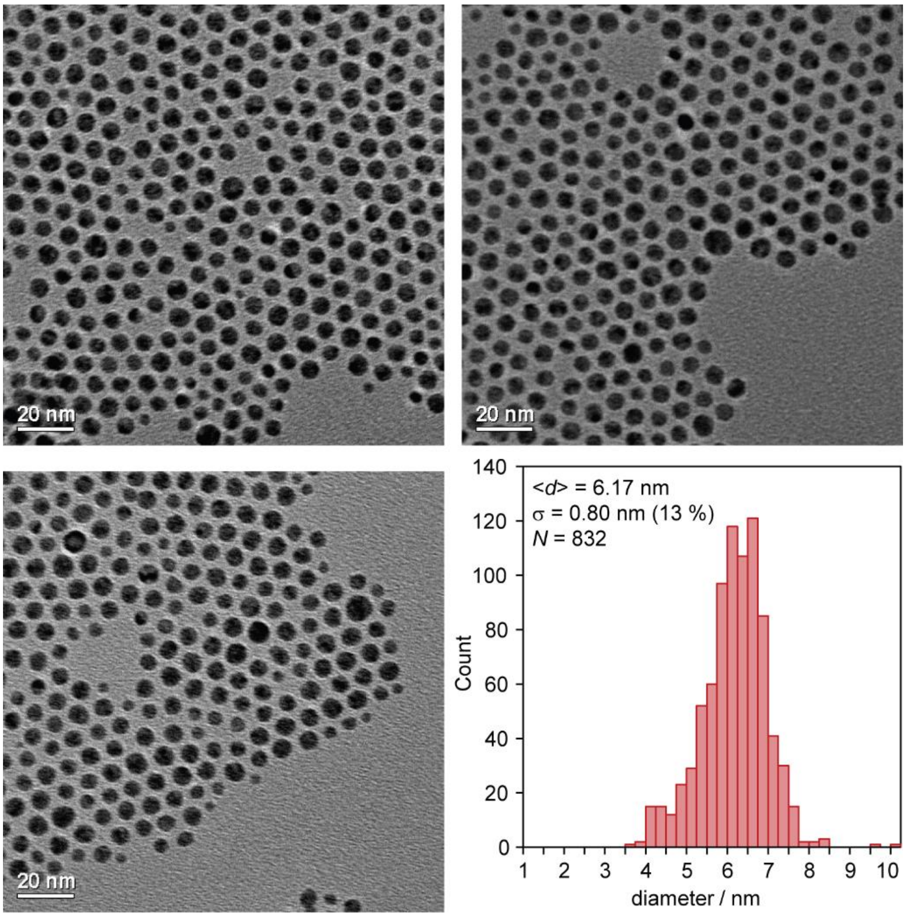

Figure S7. AuNP-**1**, toluene, slow addition over 2 h. Representative TEM images and size histogram.

AuNP-2

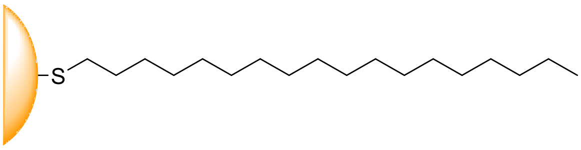

Ligand precursor: octadecanethiol (**2**)

Reaction solvent: $CHCl_3$ or toluene

Non-solvent for nanoparticle precipitation: MeOH

Slow addition time period: 2 h.

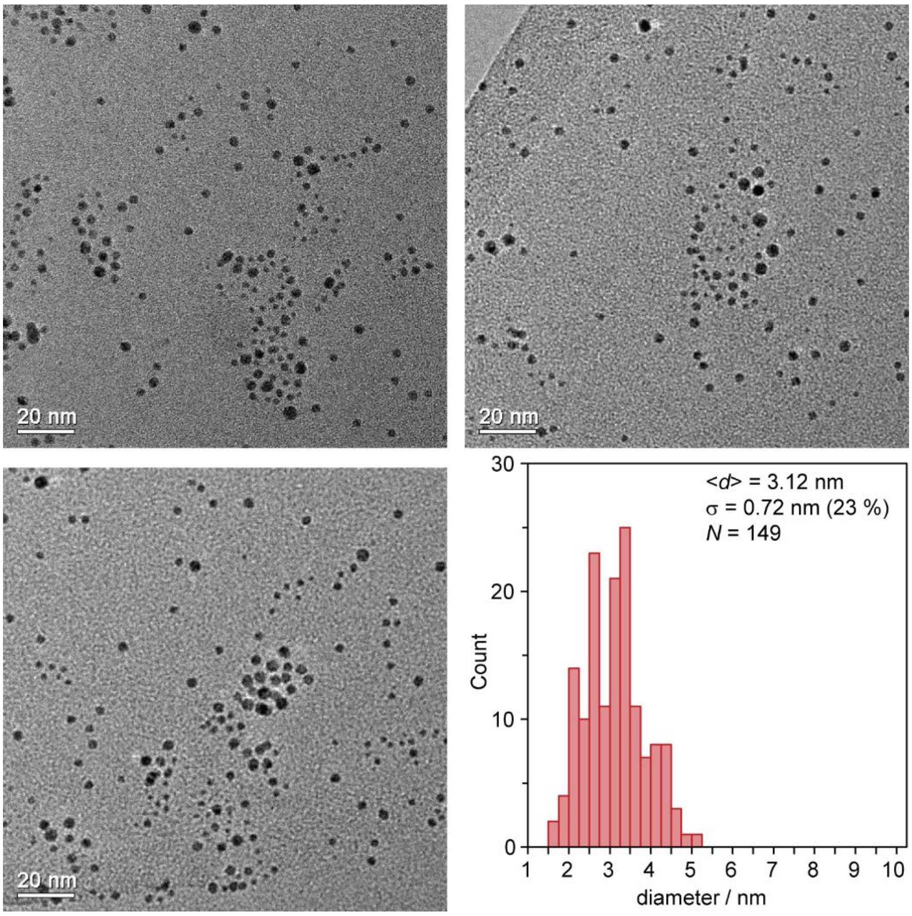

Figure S8. AuNP-**2**, $CHCl_3$, instant addition. Representative TEM images and size histogram.

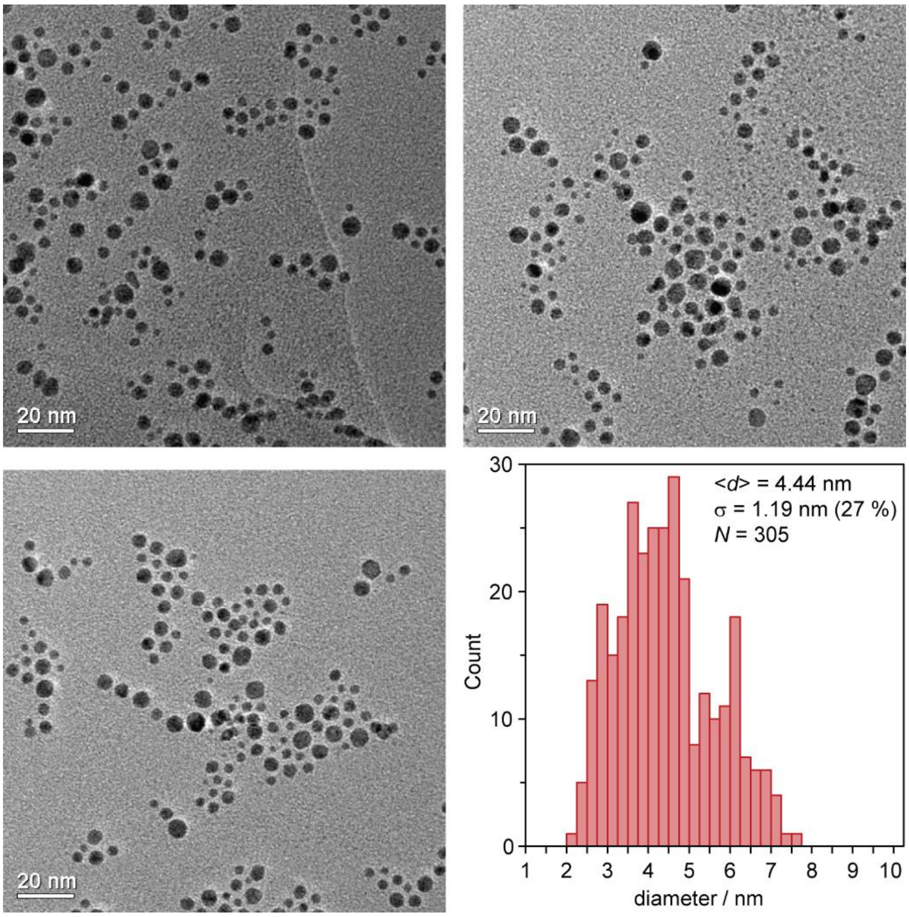

Figure S9. AuNP-**2**, $CHCl_3$, slow addition over 2 h. Representative TEM images and size histogram.

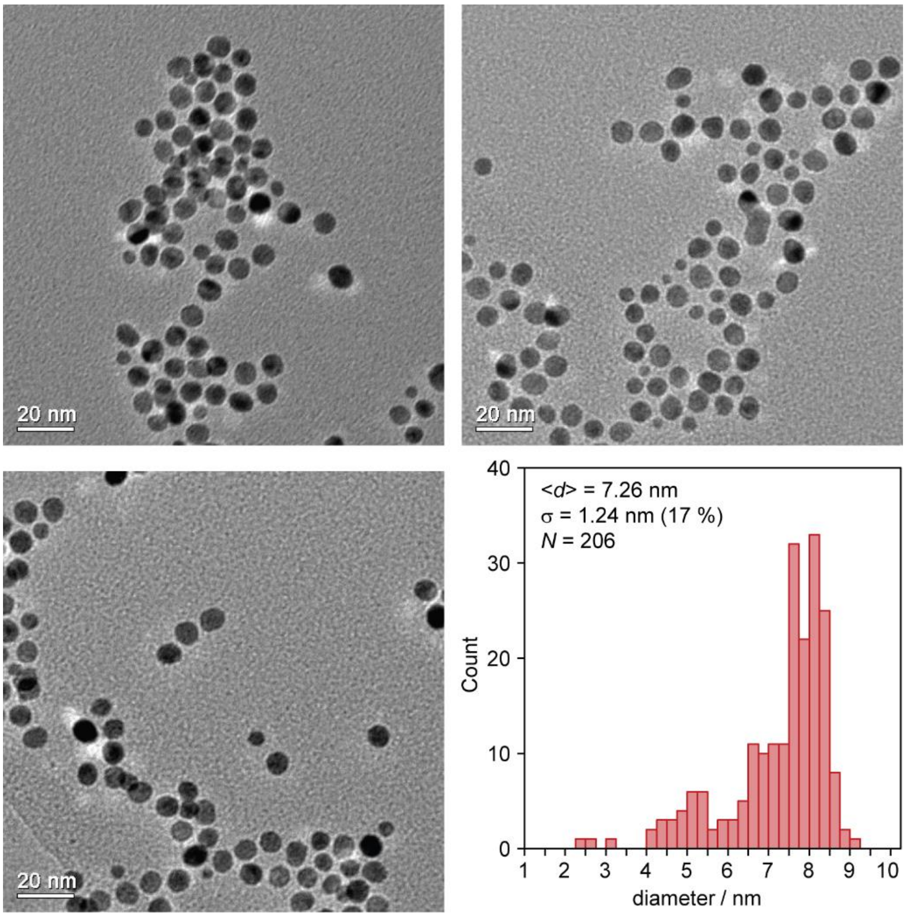

Figure S10. AuNP-**2**, toluene, instant addition. Representative TEM images and size histogram.

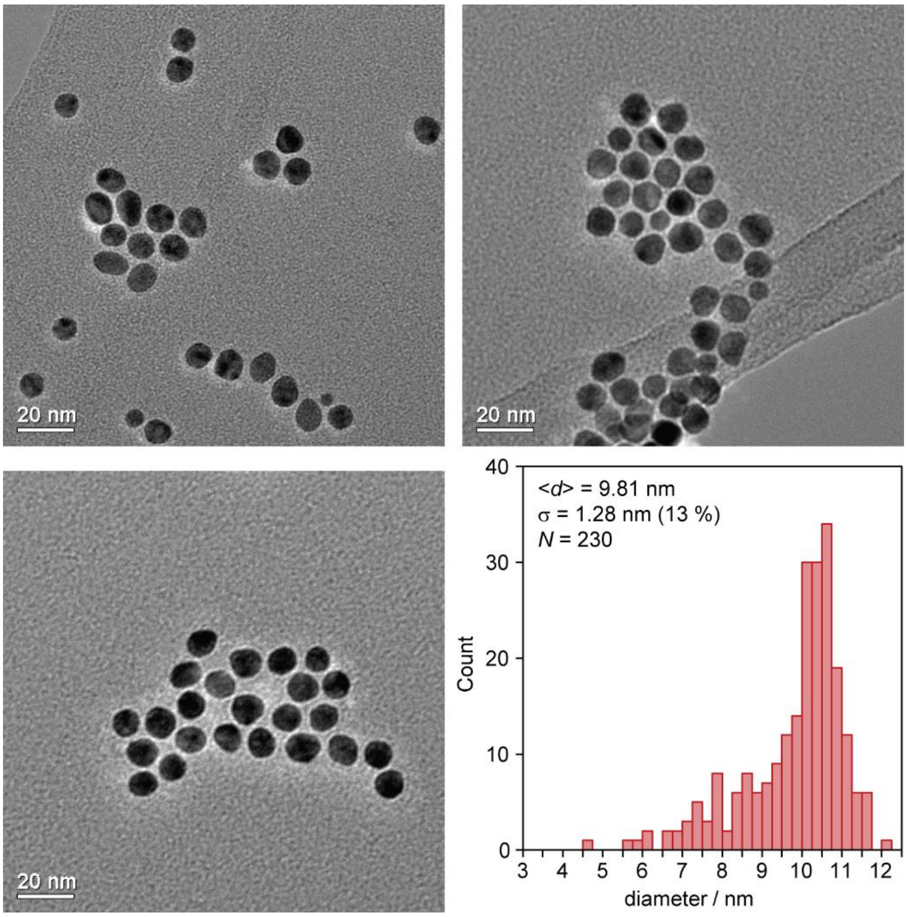

Figure S11. AuNP-**2**, toluene, slow addition over 2 h. Representative TEM images and size histogram.

AuNP-3

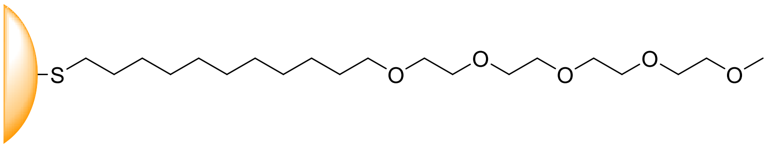

Ligand precursor: disulfide $\mathbf{3}_2$

Reaction solvent: DMF/THF 1:1 *v*/*v*

Non-solvent for nanoparticle precipitation: Et_2O/cyclohexane 1:1 *v*/*v* (instant addition); Et_2O (slow addition)

Slow addition time period: 2 h.

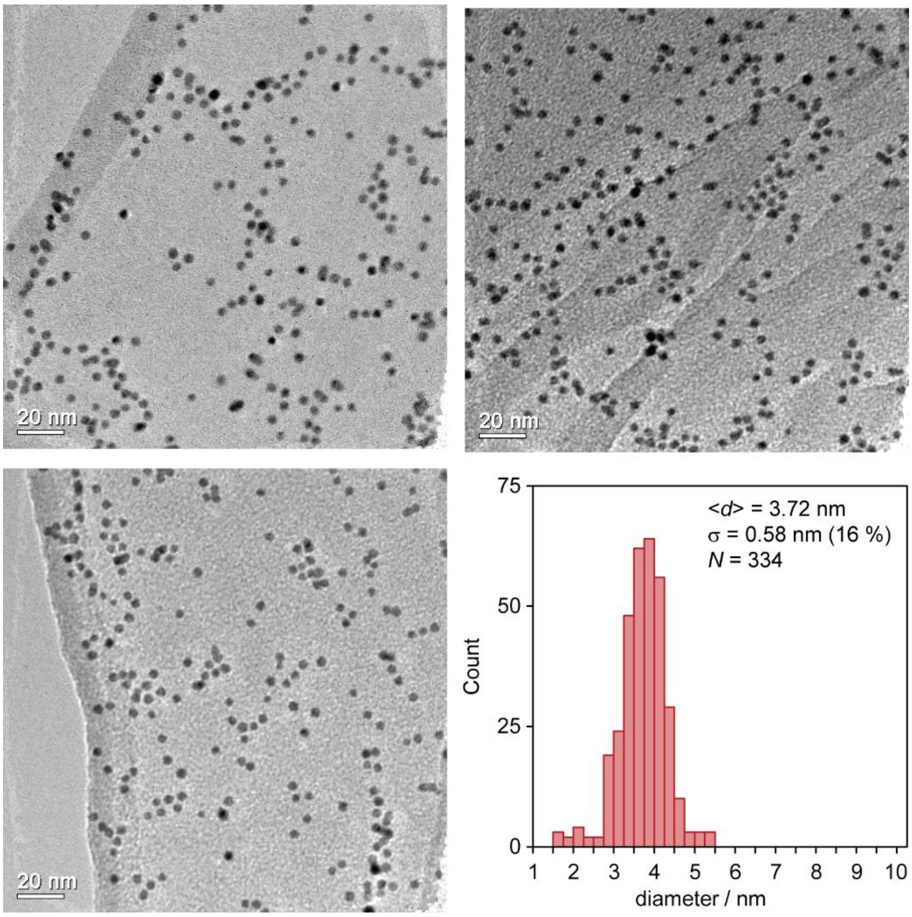

Figure S12. AuNP-**3**, instant addition. Representative TEM images and size histogram.

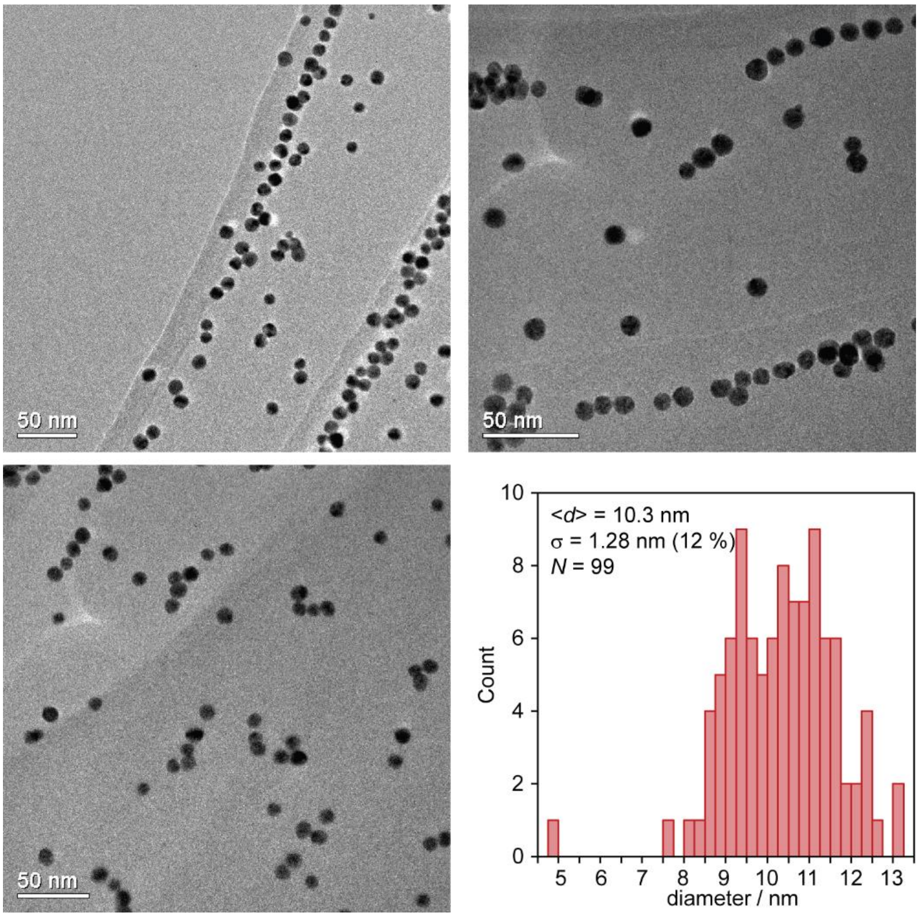

Figure S13. AuNP-**3**, slow addition over 2 h. Representative TEM images and size histogram.

AuNP-4

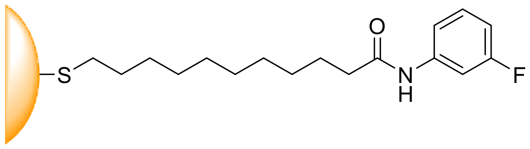

Ligand precursor: disulfide $\mathbf{4}_2$

Reaction solvent: THF/MeOH 10:1 *v*/*v*

Non-solvent for nanoparticle precipitation: water

Slow addition time period: 1 h, 2 h.

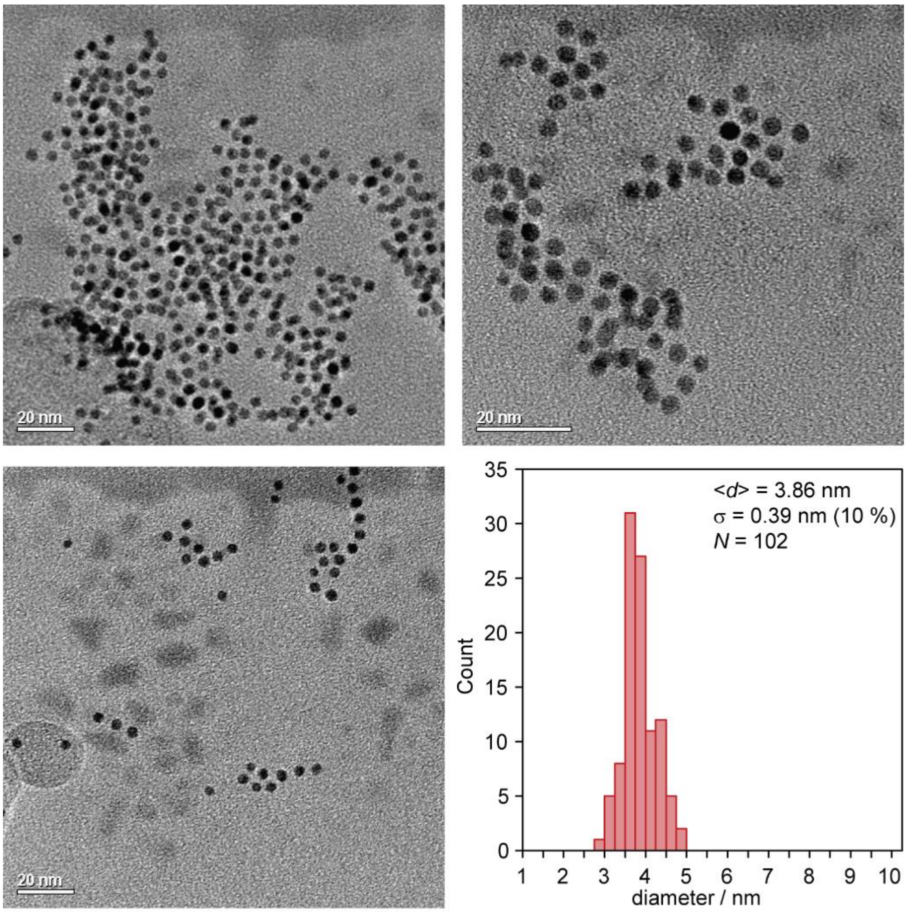

Figure S14. AuNP-**4**, instant addition. Representative TEM images and size histogram.

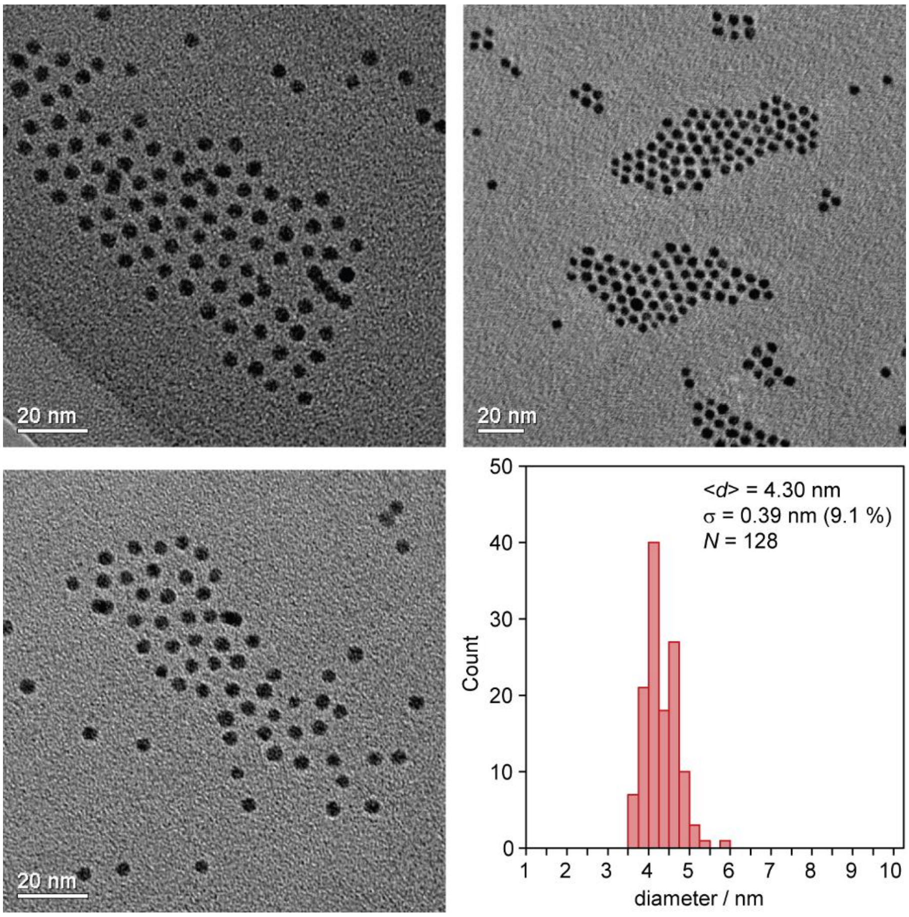

Figure S15. AuNP-**4**, slow addition over 1 h. Representative TEM images and size histogram.

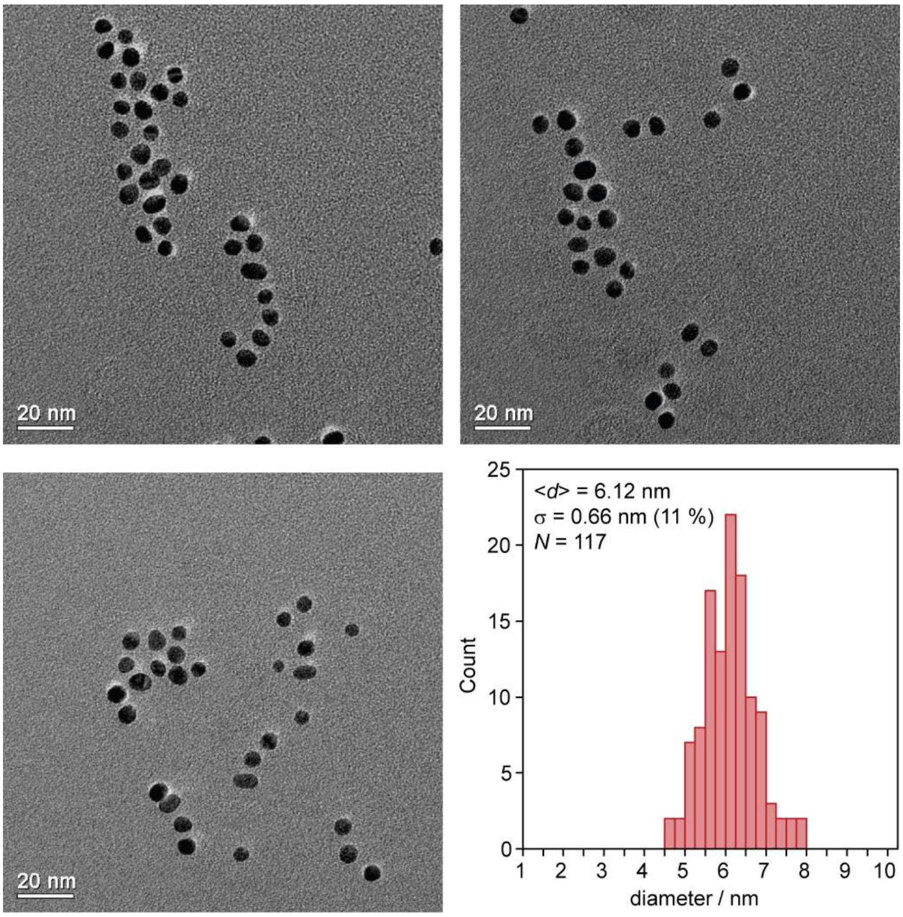

Figure S16. AuNP-**4**, slow addition over 2 h. Representative TEM images and size histogram.

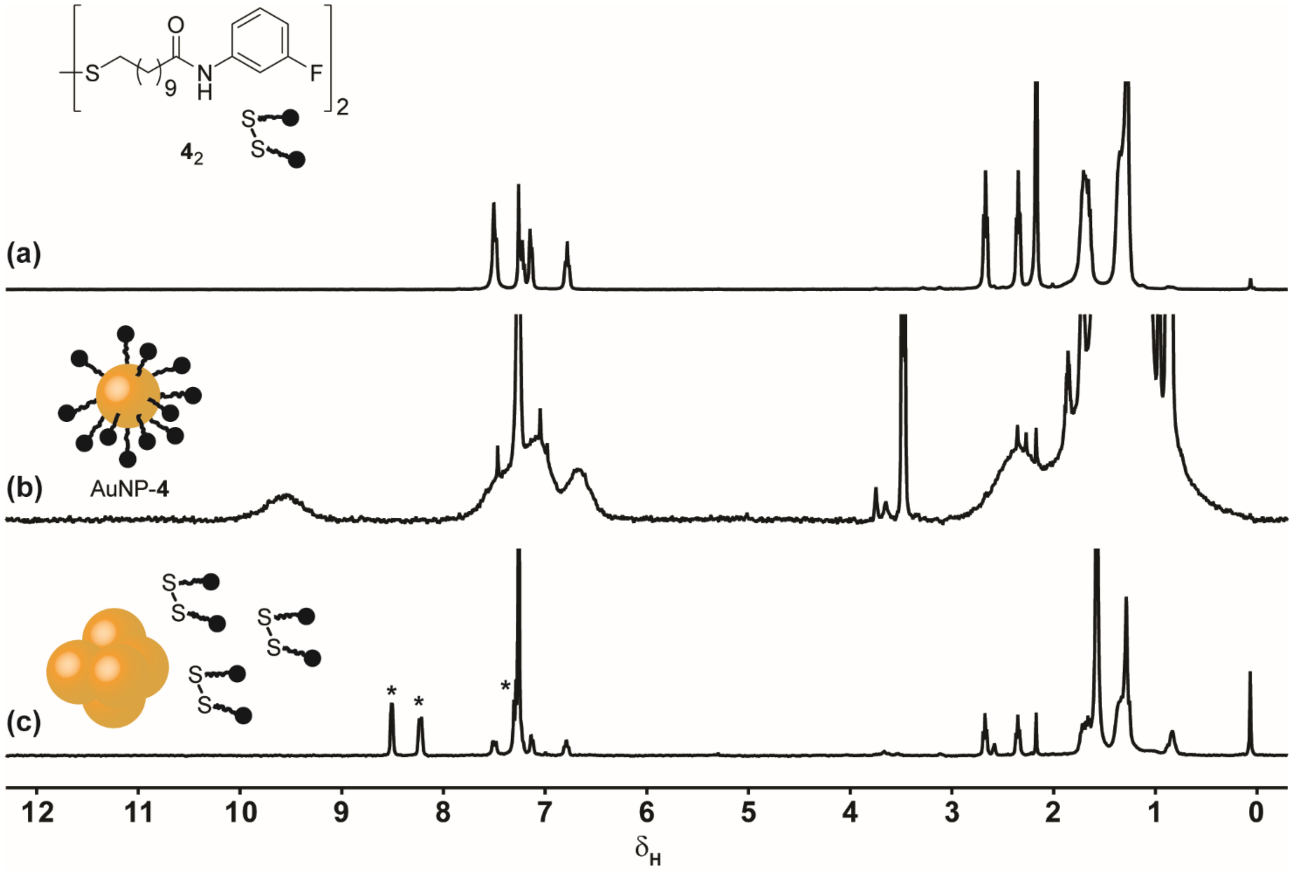

Figure S17. ^{1}H NMR spectra ($CDCl_3$) of (a) disulfide pro-ligand $\mathbf{4}_2$ (400 MHz); (b) AuNP-**4** (500 MHz); (c) supernatant following oxidative ligand desorption by treating AuNP-**4** with I_2 (400 MHz). Peaks labelled (*) correspond to 4-bromo-3-fluoronitrobenzene added as an internal standard.

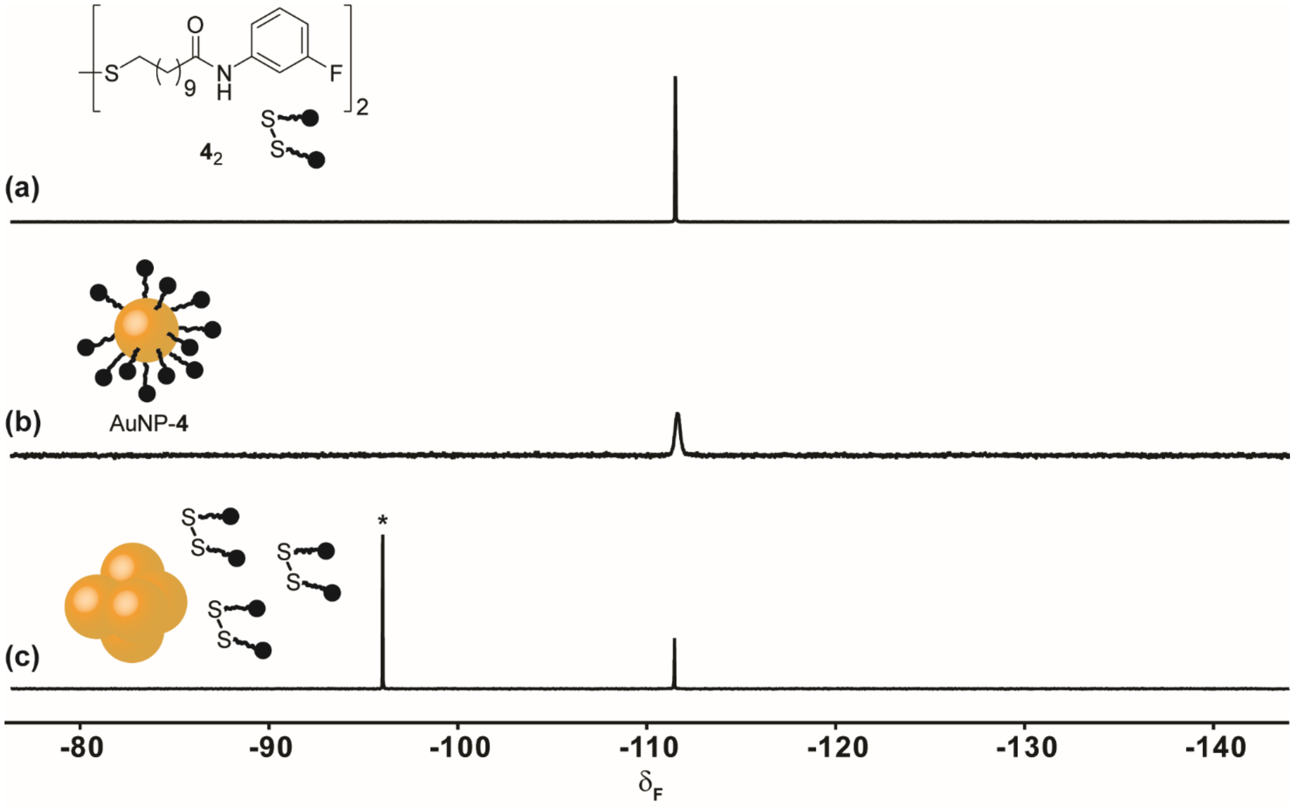

Figure S18. ^{19}F{^{1}H} NMR spectra ($CDCl_3$) of (a) disulfide pro-ligand $\mathbf{4}_2$ (376 MHz); (b) AuNP-**4** (470 MHz); (c) supernatant following oxidative ligand desorption by treating AuNP-**4** with I_2 (376 MHz). Peak labelled (*) corresponds to 4-bromo-3-fluoronitrobenzene added as an internal standard.

AuNP-5

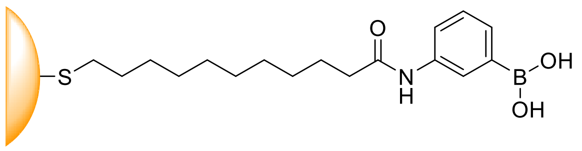

Ligand precursor: disulfide $\mathbf{5}_2$

Reaction solvent: THF/MeOH 10:1 *v*/*v*

Non-solvent for nanoparticle precipitation: Et_2O

Slow addition time period: 1 h.

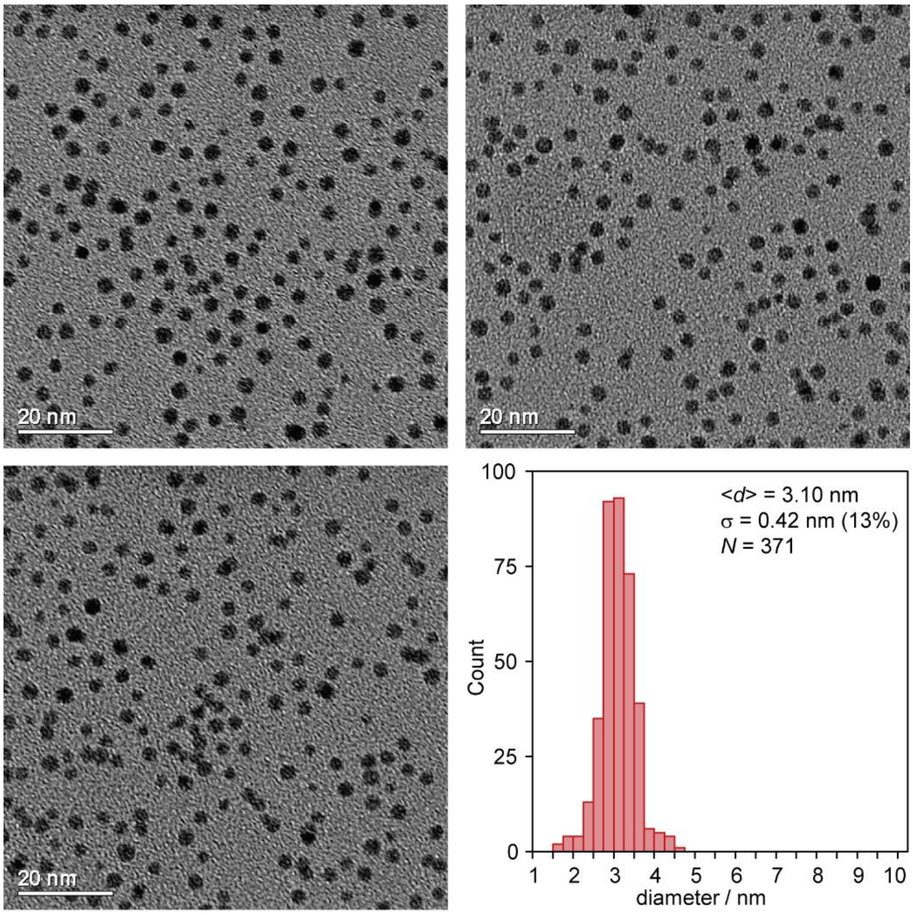

Figure S19. AuNP-**5**, instant addition. Representative TEM images and size histogram.

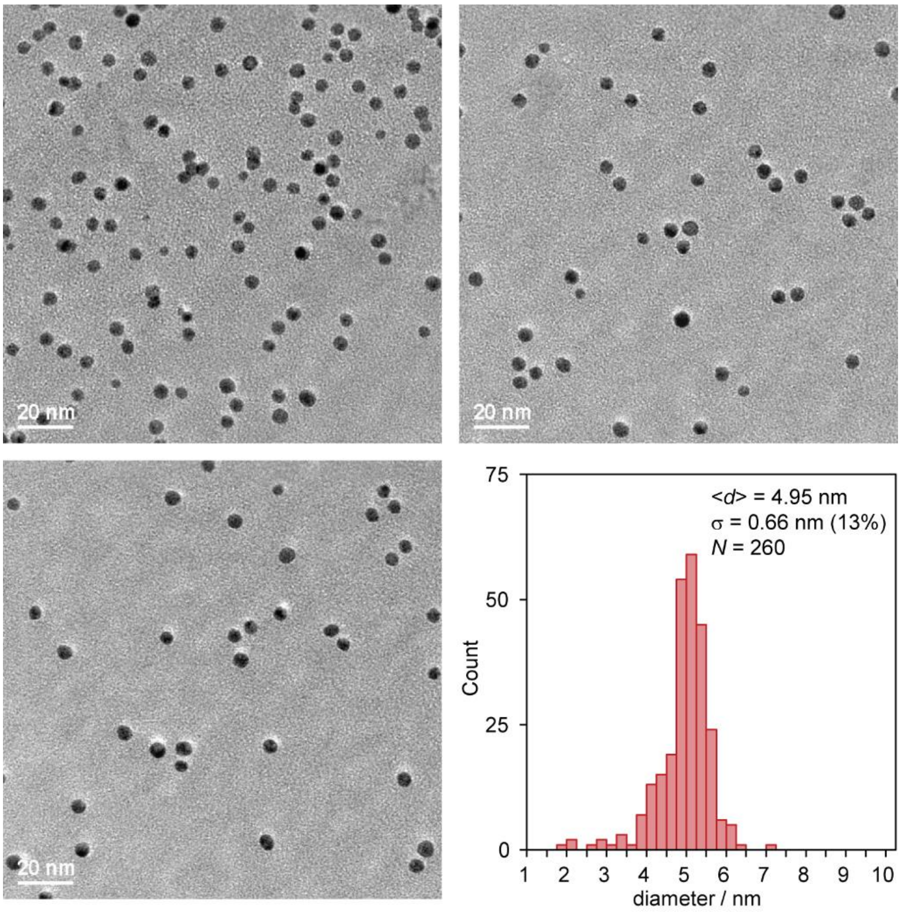

Figure S20. AuNP-**5**, slow addition over 1 h. Representative TEM images and size histogram.

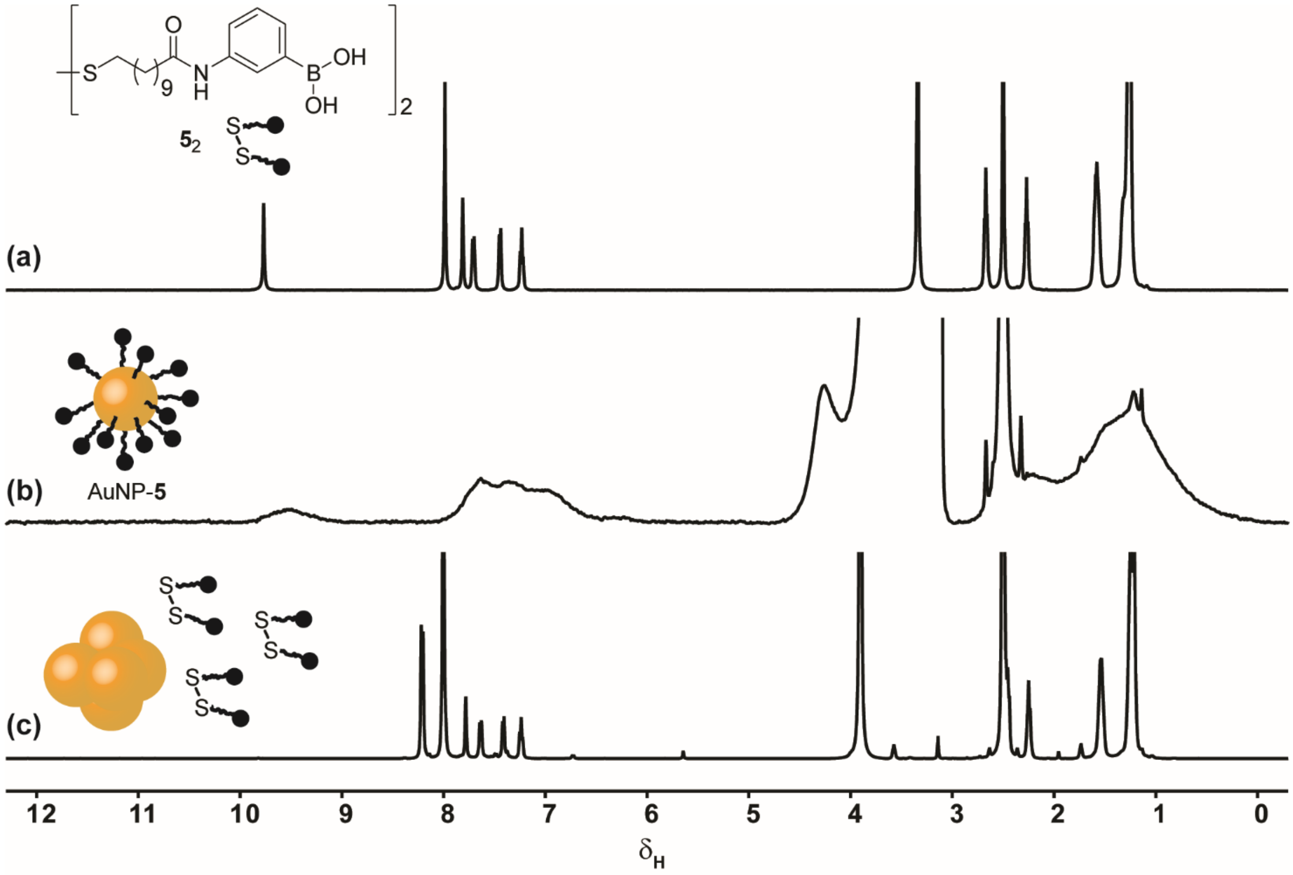

Figure S21. ^{1}H NMR spectra (DMSO-d_6) of (a) disulfide pro-ligand $\mathbf{5}_2$ (500 MHz); (b) AuNP-**5** (400 MHz); (c) supernatant following oxidative ligand desorption by treating AuNP-**5** with I_2 (500 MHz).

AuNP-6

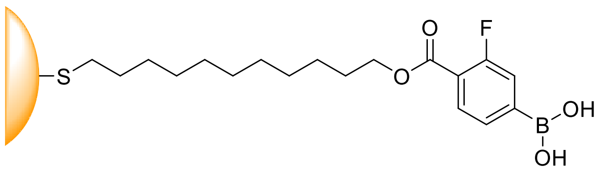

Ligand precursor: disulfide **S4**$_2$

Reaction solvent: DMF/MeOH 10:1 *v*/*v*

Non-solvent for nanoparticle precipitation: Et_2O

Slow addition time period: 2 h.

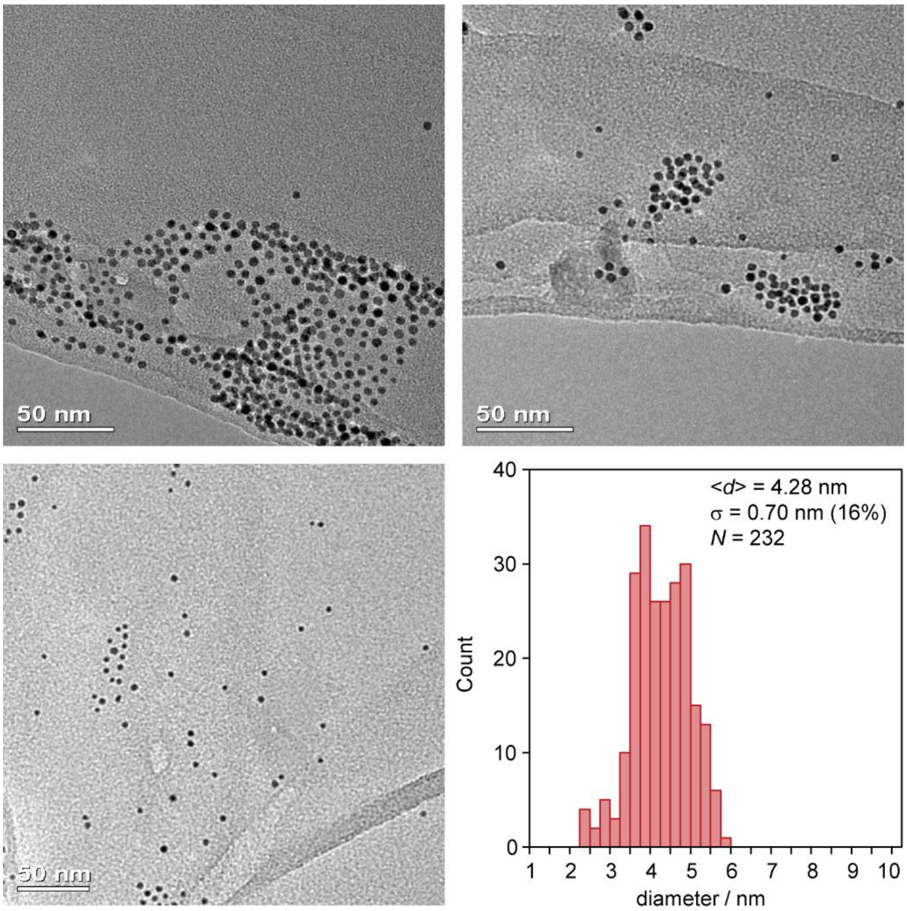

Figure S22. AuNP-**6**, instant addition. Representative TEM images and size histogram.

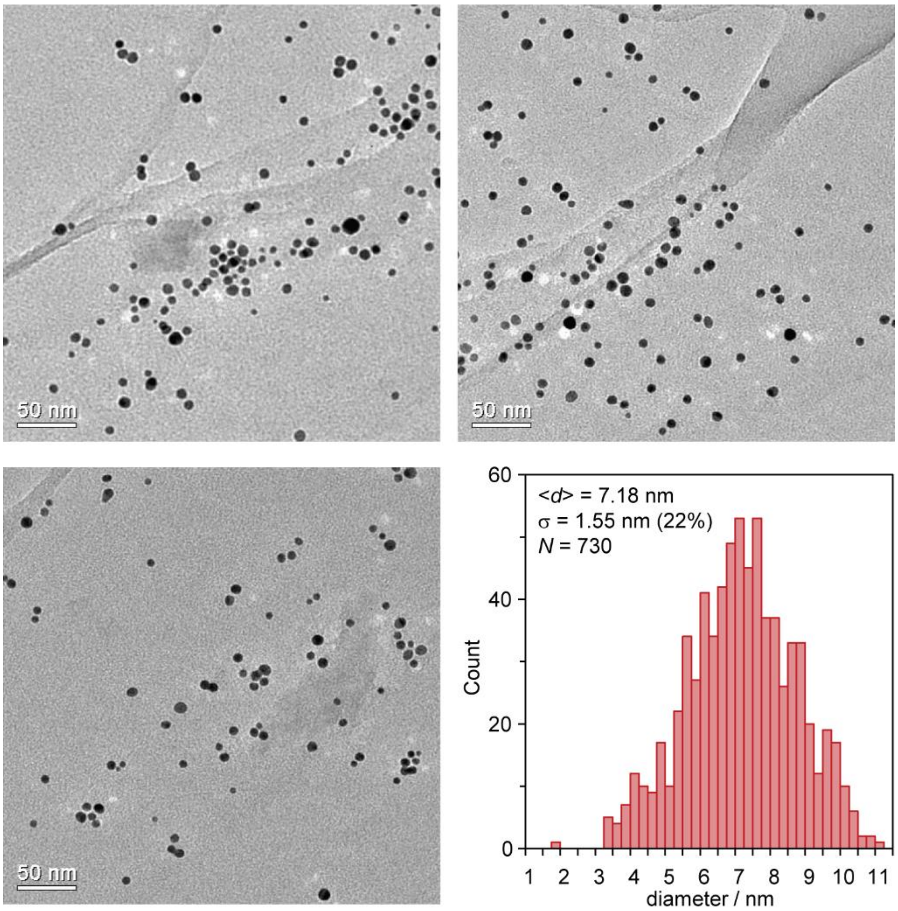

Figure S23. AuNP-**6**, slow addition over 2 h. Representative TEM images and size histogram.

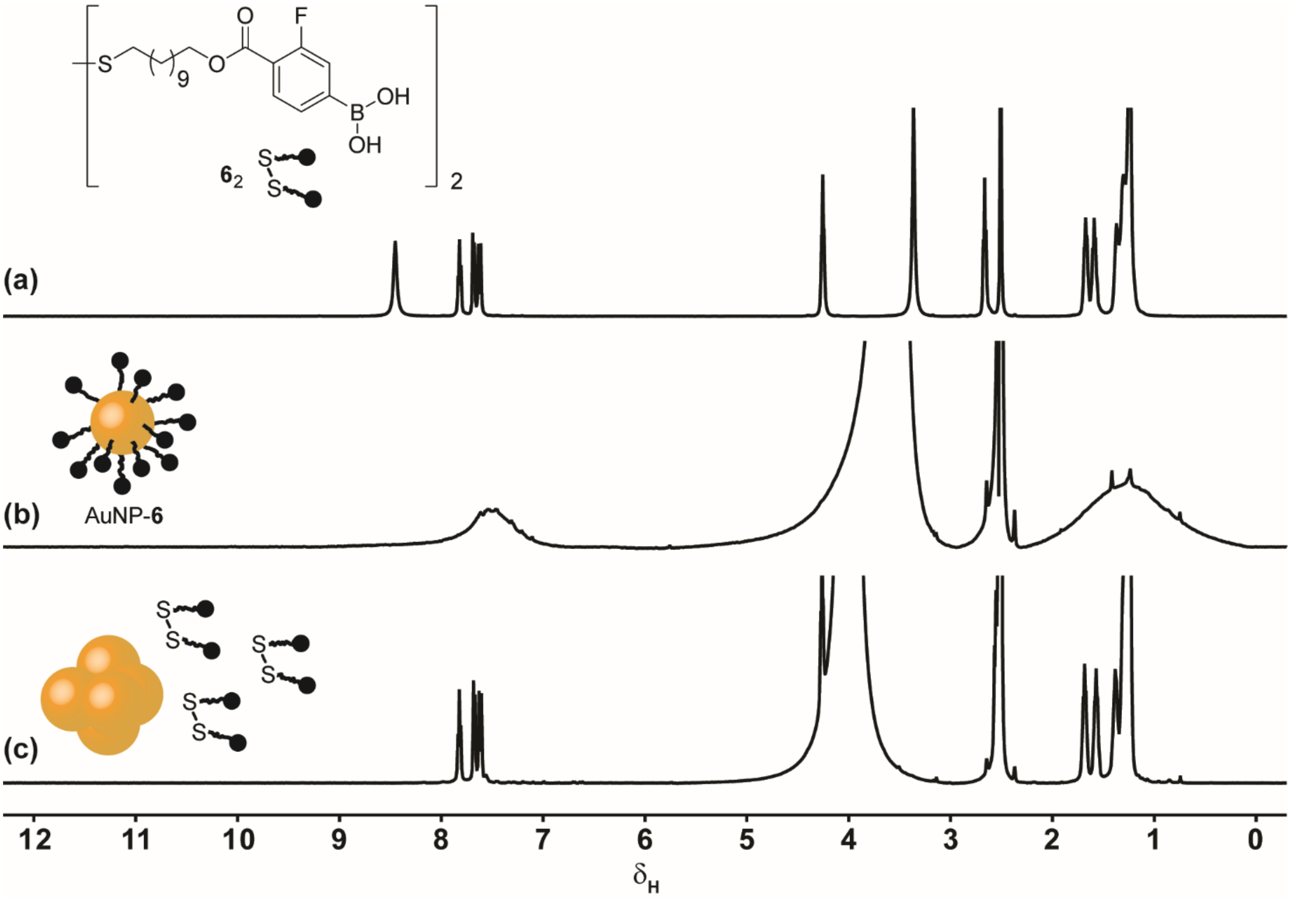

Figure S24. ^{1}H NMR spectra (DMSO-d_6, 500 MHz) of (a) disulfide **6**$_2$; (b) AuNP-**6**; (c) supernatant following oxidative ligand desorption by treating AuNP-**6** with I_2.

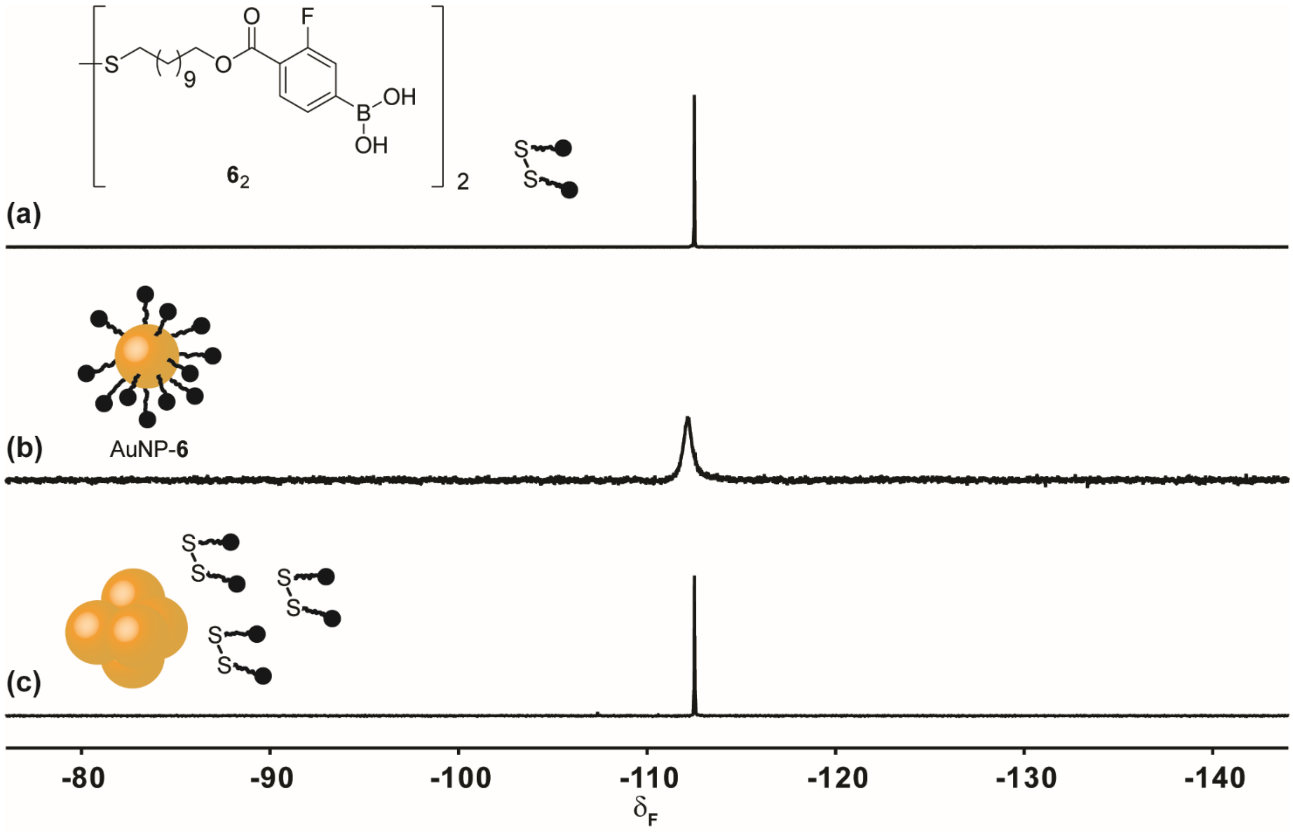

Figure S25. $^{19}F\{^{1}H\}$ NMR spectra (DMSO-*d*$_6$, 470 MHz) of (a) disulfide pro-ligand **6**$_2$; (b) AuNP-**6**; (c) supernatant following oxidative ligand desorption by treating AuNP-**6** with I_2.

AuNP-7

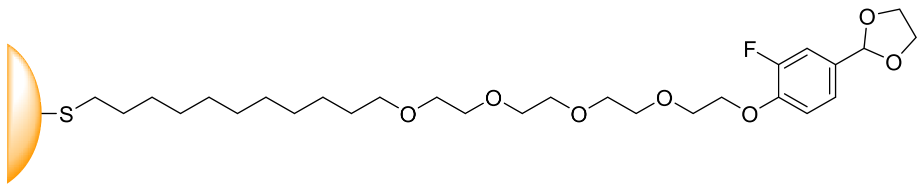

Ligand precursor: disulfide **7**$_2$

Reaction solvent: THF/DMF 9:1 *v*/*v*

Non-solvent for nanoparticle precipitation: Et_2O

Slow addition time period: 0.5 h.

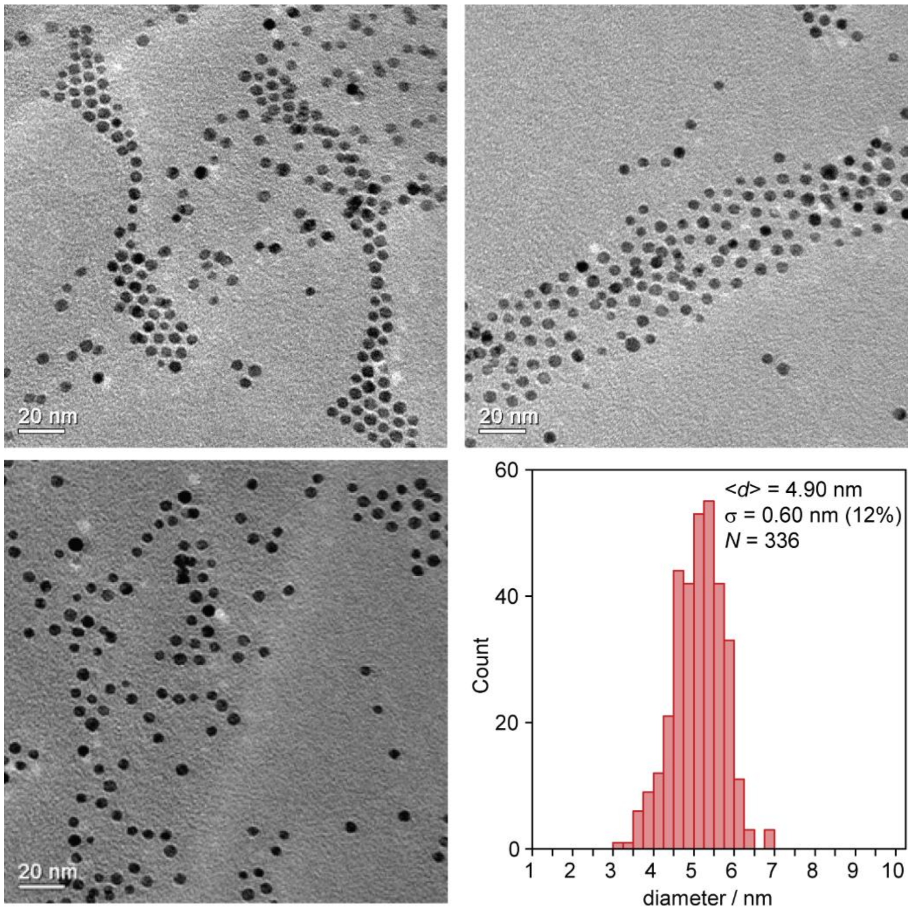

Figure S26. AuNP-**7**, instant addition. Representative TEM images and size histogram.

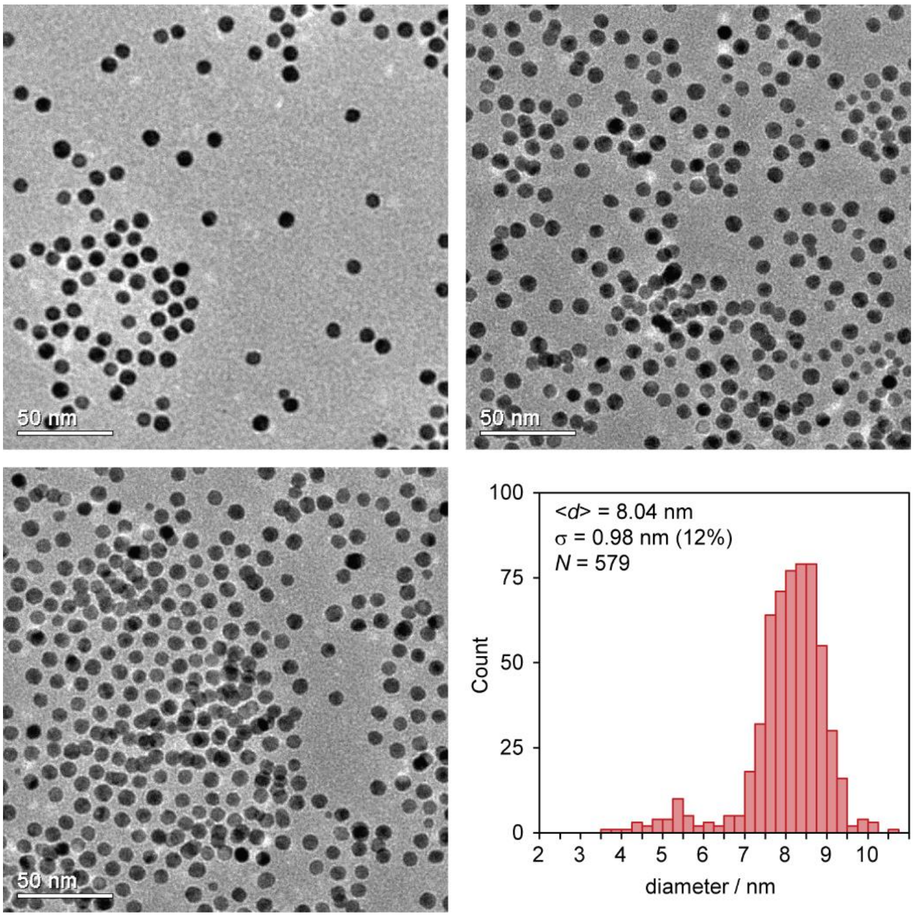

Figure S27. AuNP-**7**, slow addition over 0.5 h. Representative TEM images and size histogram.

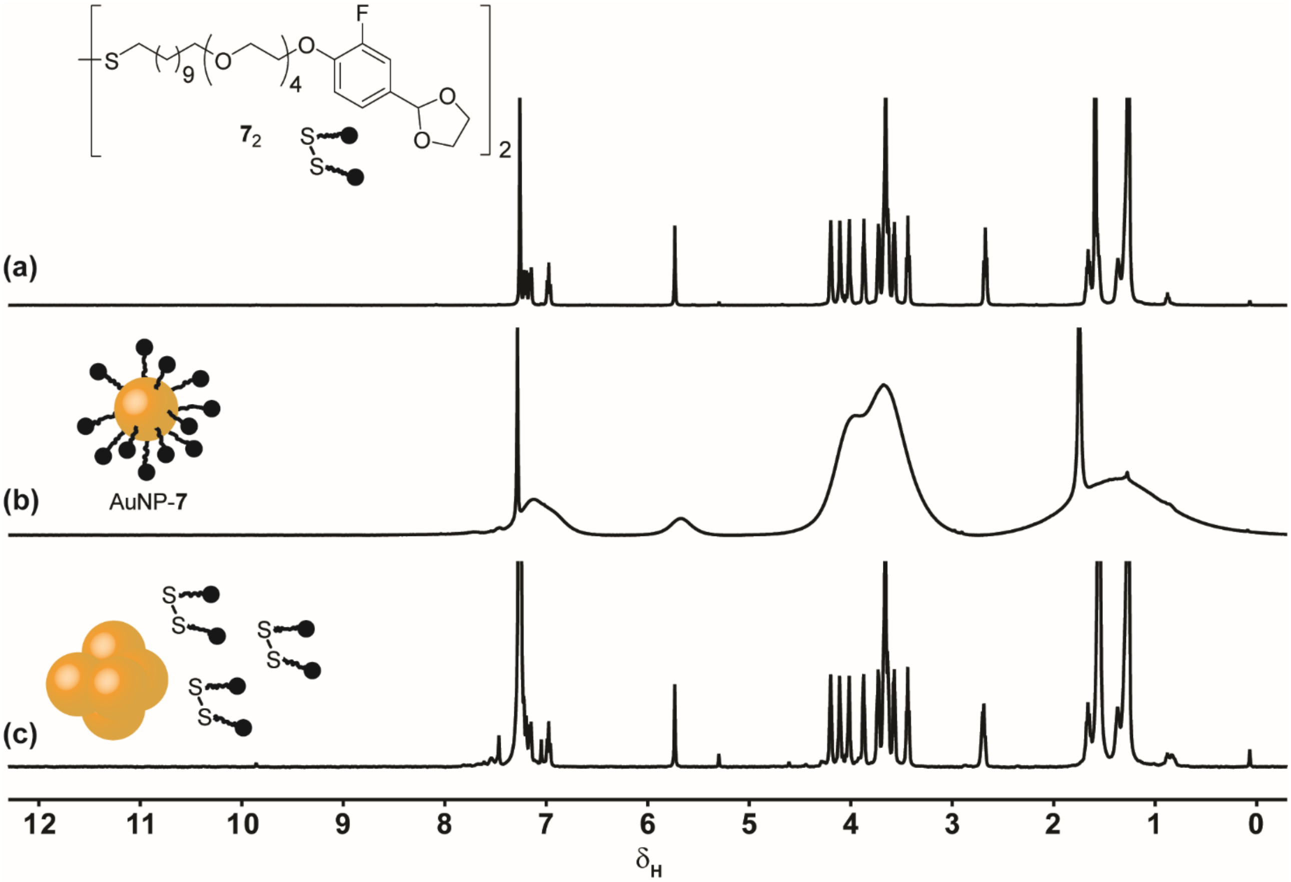

Figure S28. ^{1}H NMR spectra ($CDCl_3$) of (a) disulfide pro-ligand $\mathbf{7}_2$ (500 MHz); (b) AuNP-**7** (400 MHz); (c) supernatant following oxidative ligand desorption by treating AuNP-**7** with I_2 (500 MHz).

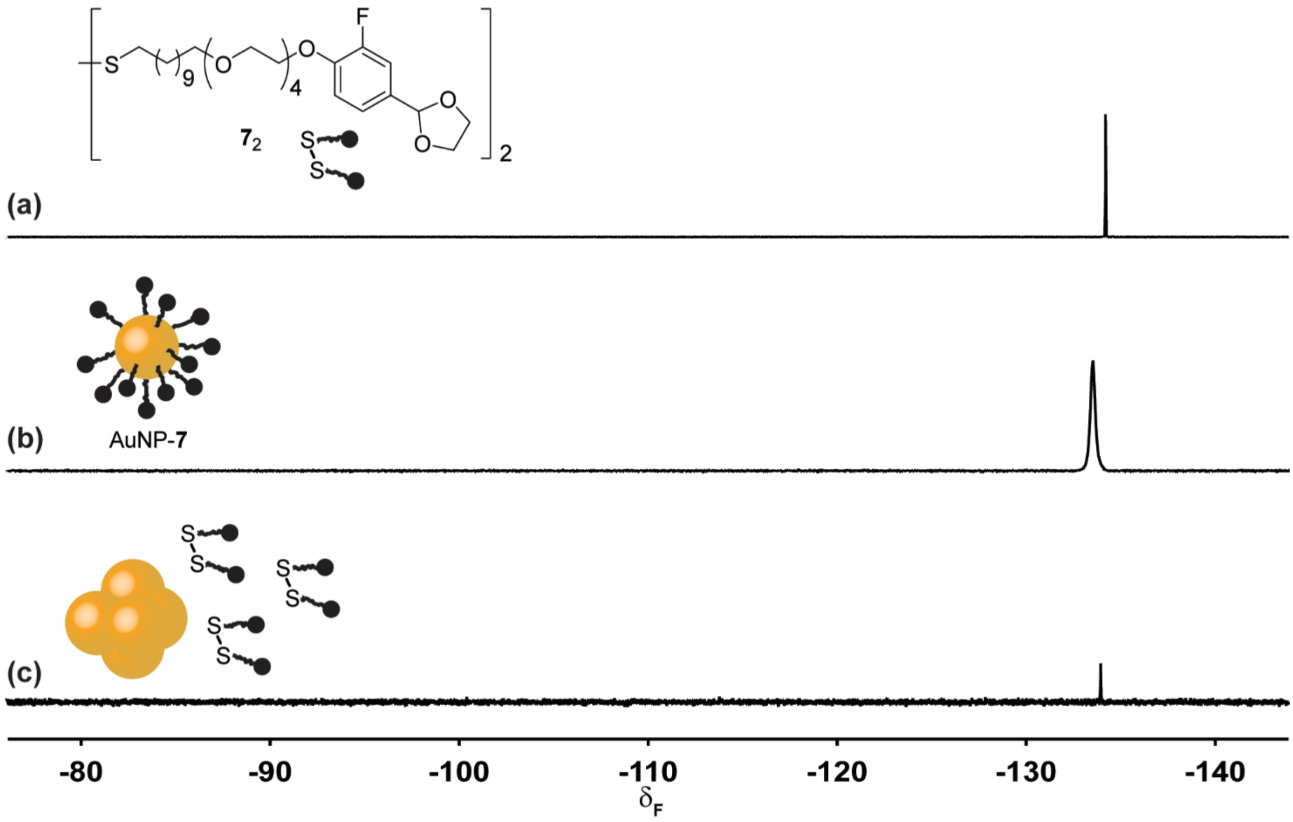

Figure S29. $^{19}F\{^1H\}$ NMR spectra ($CDCl_3$) of (a) disulfide pro-ligand **7**$_2$ (470 MHz); (b) AuNP-**7** (376 MHz); (c) supernatant following oxidative ligand desorption by treating AuNP-**7** with I_2 (470 MHz).

4. References

(1) della Sala, F.; Kay, E. R. Reversible Control of Nanoparticle Functionalization and Physicochemical Properties by Dynamic Covalent Exchange. *Angew. Chem. Int. Ed.* **2015**, *54*, 4187-4191.
(2) Borsley, S.; Kay, E. R. Dynamic Covalent Assembly and Disassembly of Nanoparticle Aggregates. *Chem. Commun.* **2016**, *52*, 9117-9120.
(3) Yang, Y.; Poss, G.; Weng, Y. N.; Qi, R. Z.; Zheng, H. R.; Nianias, N.; Kay, E. R.; Guldin, S. Probing the Interaction of Nanoparticles with Small Molecules in Real Time Via Quartz Crystal Microbalance Monitoring. *Nanoscale* **2019**, *11*, 11107-11113.
(4) Diez-Castellnou, M.; Suo, R.; Marro, N.; Matthew, S. A. L.; Kay, E. R. Rapidly Adaptive All-Covalent Nanoparticle Surface Engineering. *Chem. - Eur. J.* **2021**, *27*, 9948-9953.
